# Capturing spiral radial growth of conifers using the superellipse to model tree-ring geometric shape

**DOI:** 10.3389/fpls.2015.00856

**Published:** 2015-10-15

**Authors:** Pei-Jian Shi, Jian-Guo Huang, Cang Hui, Henri D. Grissino-Mayer, Jacques C. Tardif, Li-Hong Zhai, Fu-Sheng Wang, Bai-Lian Li

**Affiliations:** ^1^Co-Innovation Centre for Sustainable Forestry in Southern China, Bamboo Research Institute, Nanjing Forestry UniversityNanjing, China; ^2^Key Laboratory of Vegetation Restoration and Management of Degraded Ecosystems, South China Botanical Garden, Chinese Academy of SciencesGuangzhou, China; ^3^Provincial Key Laboratory of Applied Botany, South China Botanical Garden, Chinese Academy of SciencesGuangzhou, China; ^4^Department of Mathematical Sciences, Centre for Invasion Biology, Stellenbosch UniversityMatieland, South Africa; ^5^Mathematical and Physical Biosciences, African Institute for Mathematical SciencesCape Town, South Africa; ^6^Department of Geography, The University of TennesseeKnoxville, TN, USA; ^7^Centre for Forest Interdisciplinary Research, University of WinnipegWinnipeg, MB, Canada; ^8^Ecological Complexity and Modelling Laboratory, Department of Botany and Plant Sciences, University of California, RiversideRiverside, CA, USA

**Keywords:** basal area, cross section, major semi-axis, polar coordinate, rotation, tree-rings

## Abstract

Tree-rings are often assumed to approximate a circular shape when estimating forest productivity and carbon dynamics. However, tree rings are rarely, if ever, circular, thereby possibly resulting in under- or over-estimation in forest productivity and carbon sequestration. Given the crucial role played by tree ring data in assessing forest productivity and carbon storage within a context of global change, it is particularly important that mathematical models adequately render cross-sectional area increment derived from tree rings. We modeled the geometric shape of tree rings using the superellipse equation and checked its validation based on the theoretical simulation and six actual cross sections collected from three conifers. We found that the superellipse better describes the geometric shape of tree rings than the circle commonly used. We showed that a spiral growth trend exists on the radial section over time, which might be closely related to spiral grain along the longitudinal axis. The superellipse generally had higher accuracy than the circle in predicting the basal area increment, resulting in an improved estimate for the basal area. The superellipse may allow better assessing forest productivity and carbon storage in terrestrial forest ecosystems.

## Introduction

Tree rings are natural archives of environmental changes and they have long been used in exploring the effects of endogenous (e.g., competition) and exogenous (e.g., climate, disturbances) factors on tree growth (Fritts, 1976). For example, tree-ring data have been widely used in climate reconstructions (Cook et al., 2002; Frank and Esper, 2005), disturbance reconstructions (Bergeron et al., 2004; Stoffel and Corona, 2014), investigation of species competition and succession (Callaway, 1998; Linares et al., 2010; Huang et al., 2013), and assessments of forest carbon storage and equilibrium (Guyette et al., 2008; Davis et al., 2009; Ma et al., 2012).

Traditionally forest sciences including tree-ring techniques often have assumed that tree rings on a cross section approximate a series of concentric circles (Biondi and Qeadan, 2008; West, 2009). Based on this assumption, mean annual ring-width and basal area increment are usually obtained and commonly used as two basic parameters for investigating environmental effects on growth and for assessing forest growth, productivity, and carbon sequestration. Mean annual ring-width is often calculated from two radial growth measurements along two directions with an angle of between 90° and 180° on a cross section collected at diameter at breast height (DBH). Basal area increment is calculated from the difference in area encircled by two adjacent rings (e.g., Biondi and Qeadan, 2008; Huang et al., 2013, 2014).

In many cases, however, tree rings can be better depicted by ellipses rather than circles, or are more inclined to be elliptical around a common centre. This geometric shape of the tree-ring boundary (thereafter referred to as tree-ring shape) has been ignored in practice given that the bias between the circular and ellipse is assumed to be small (West, 2009) although tree rings have long been used in multidisciplinary ecological research for nearly 75 years. Gielis (2003a,b) introduced a superellipse equation that can capture a wide range of geometric shapes in nature. The superellipse equation is a generalization of the traditional ellipse equation and can even produce the outline of a rectangle under special parameter values. If a tree ring can be better fitted by a superellipse function which bears a major axis and a minor axis, the following hypotheses then need to be further tested. First, because the tree trunk does not often look perfectly round, radial growth of trees may follow a spiral growth pattern over time on the cross section in contrast to spiral grain over the longitudinal axis (see the following section below), such that the direction of the major axis of the superellipse may vary with age. Second, basal area increment can be better estimated using the superellipse equation than the circle equation.

Spiral grain is a growth phenomenon in trees characterized by a helical structure of fibers around the pith rather than a longitudinal structure of fibers along the stem axis (Skatter and Kucera, 1998). Spiral grain along the longitudinal axis has been widely observed and reported in many coniferous and some broadleaf species (Harris, 1989; Kubler, 1991; Skatter and Kucera, 1998; Wing et al., 2014). Supplementary Figure 1 exhibits an example of spiral grain of dragon juniper [*Sabina chinensis* (L.) Ant. cv. Kaizuca]. A general agreement is that many tree species (particularly conifers) usually develop a left-handed (L) spirality (when viewed from below) while young. The grain angle then shifts gradually toward right-handed (R) spirality, ending up with a remarkable right-handed grain angle during the mature stage (Skatter and Kucera, 1998). This is the “LR” pattern commonly observed (Harris, 1989) while the opposite pattern (“RL”) has also been proposed but only for fewer tree species (Balodis, 1972; Harris, 1989; Harding and Woolaston, 1991). The LR or RL pattern is widely believed to be controlled strongly by genetic factors and less by environmental factors, such as strong wind or water shortage that dominates on one side of the tree (Kubler, 1991; Gapare et al., 2009; Wing et al., 2014). For example, Wing et al. (2014) found no correlation between spiral grain in bristlecone pines (*Pinus longaeva* D.K. Bailey) and environmental factors. In contrast, spiral growth on the radial section was previously acknowledged (Kubler, 1991) but has never been thoroughly investigated and understood. Knowledge on spiral growth on the radial section obtained through model fit with the superellipse may help better understand the long-term debate on spiral growth over the longitudinal section mentioned above, which is closely related to wood quality and forest productivity. Consequently they may together contribute to an improved estimation of growth of trees and forests, as well as for carbon storage and equilibrium of terrestrial forest ecosystems, and ultimately sustainable forest management within the context of global change.

In this study, we attempt to: (1) use the superellipse equation to model tree-ring shapes of conifers which usually bear clear annual ring growth pattern; and (2) explore whether any spiral growth exists along the radial section over time and, if it does, determine whether it is related to spiral grain over the longitudinal axis.

## Materials and methods

### Superellipse equation

The superellipse equation is a generalized ellipse equation that can produce the circle, ellipse, square, and rectangle (Gielis, 2003a,b):

(1) $$\begin{array}{l} |\frac{x}{a}{|}^{n}+|\frac{y}{b}{|}^{n}=1 \end{array}$$

where *x* and *y* represent the Cartesian coordinates; *a* represents the major semi-axis radius; *b* (0 < *b* ≤ *a*) represents the minor semi-axis radius; and *n* is a power. It can also be formulated using the polar coordinates (a transformation using *x* = *r* cosφ and *y* = *r* sinφ; Gielis, 2003a,b):

(2) $$\begin{array}{l} r={\left(|\frac{\cos\phi }{a}{|}^{n}+|\frac{\sin\phi }{b}{|}^{n}\right)}^{-1∕n} \end{array}$$

where *r* represents the radial distance between the pole and a point on the boundary, and φ the angle of the radial vector. The superellipse equation becomes a typical ellipse equation when *n* = 2. Let *k = b/a* ≤ 1, and Equation (2) can be rewritten as:

(3) $$\begin{array}{l} r=a\cdot {\left(|\cos\phi {|}^{n}+|\frac{\sin\phi }{k}{|}^{n}\right)}^{-1∕n} \end{array}$$

**Figure 1:**
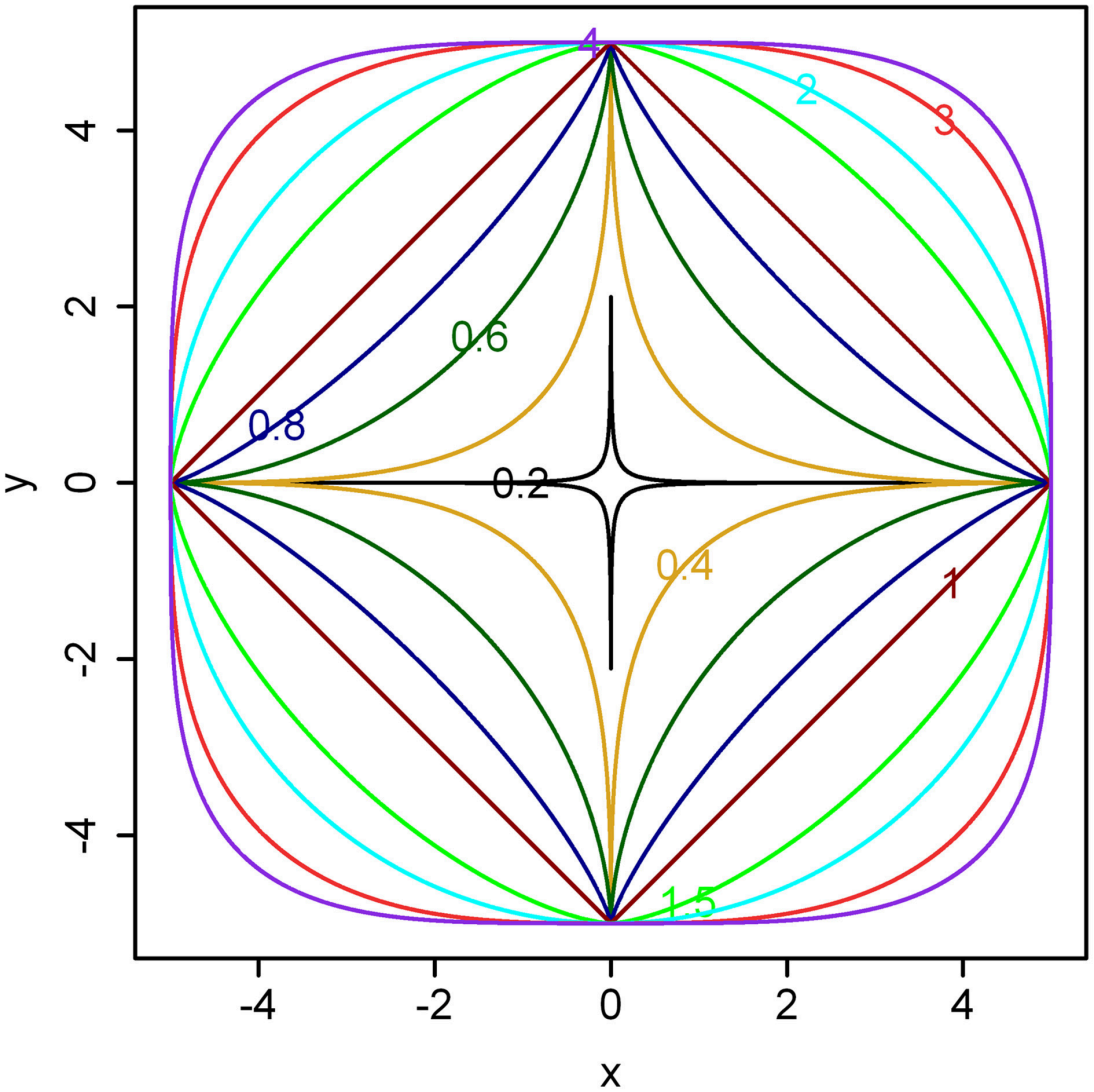
**Illustration of the superellipse equation**. Here, *a* = *b* = 5 and different powers (i.e., values for *n*) are shown in the boundaries. As the value of *n* becomes larger, the boundary gradually approximates a square. If *a* ≠ *b*, the boundary gradually approximates a rectangle.

Examples for different *n* ranging from 0.2 to 4 are illustrated in Figure 1.

### Parametric fitting

**Figure 2:**
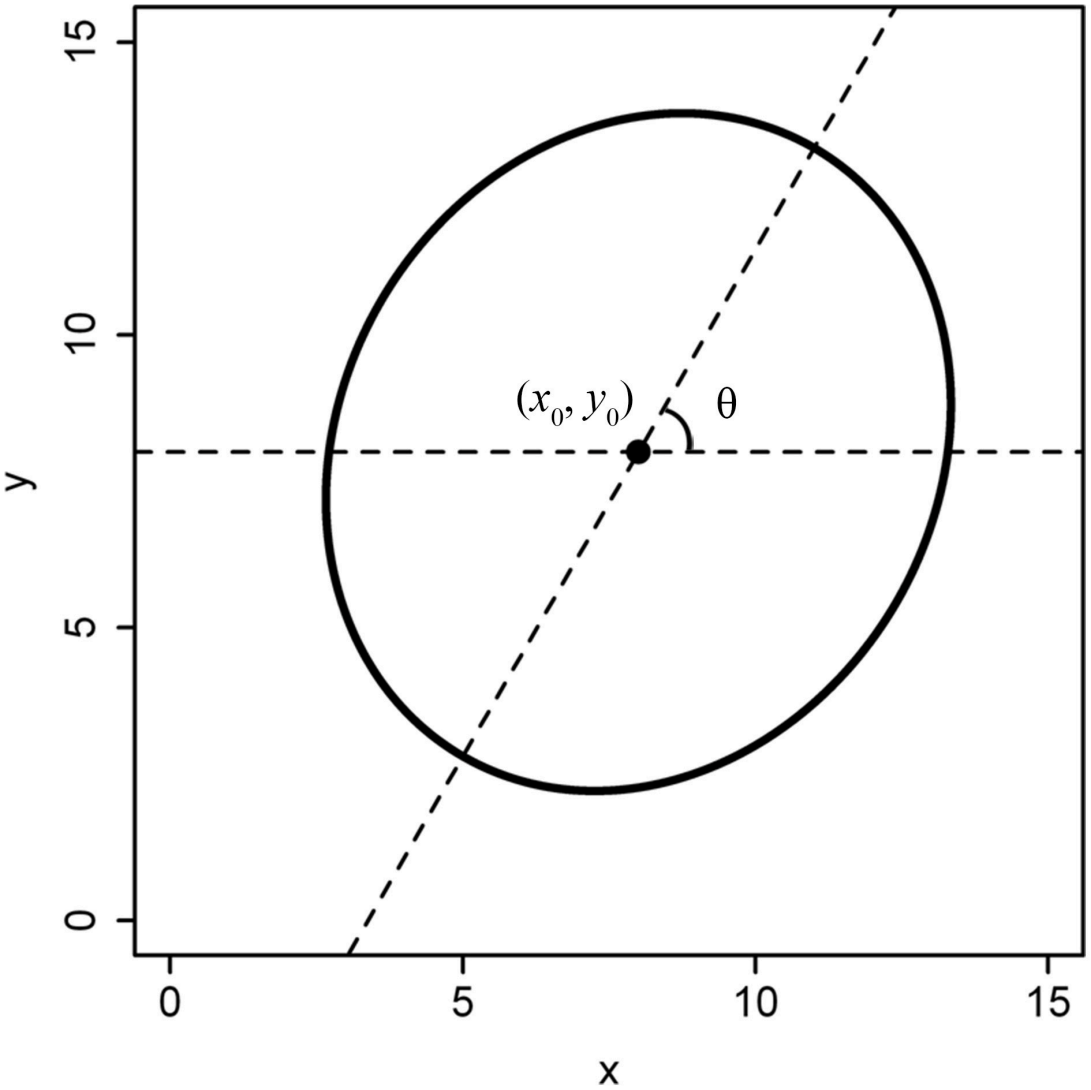
**Non-standard format of a superellipse**. The superellipse center is not the point of (0, 0), and the angle between the major axis and the horizontal axis (i.e., the x-axis) isn't equal to 0.

We define a standard superellipse equation: the origin coordinate is (0, 0), and the major axis is aligned with the horizontal axis. However, the planar coordinates of a tree ring are usually extracted from a scanned image, with the centre not exactly in the origin and the major axis not aligned with the horizontal axis (Figure 2). In this case, we refer to such a shape as a non-standard superellipse and its equation as the non-standard superellipse equation. To fit the parameters of a non-standard superellipse equation, we need first to transform the boundary coordinates to the standard format of the superellipse. Let *x*_1_ = *x*′ – *x*_0_, *y*_1_ = *y*′ – *y*_0_. Here, *x*′ and *y*′ are the *x*- and *y*-coordinates extracted from a non-standard format of the superellipse; (*x*_0_, *y*_0_) is the coordinate of the superellipse centre (i.e., the pole). Let φ′ be the angle coordinate corresponding to the point of (*x*_1_, *y*_1_) in a non-standard superellipse boundary. Obviously, φ′ = arctan(*y*_1_/*x*_1_). Let θ be the angle between the major axis in the non-standard superellipse equation and the horizontal axis. Then the angle coordinate (φ) in the standard superellipse equation is φ = φ′−θ. Then we have:

(4) $$\{\begin{array}{l} x=x_{1}\cos\;\theta +y_{1}\sin\;\theta \\ y=y_{1}\cos\;\theta -x_{1}\sin\;\theta \end{array}$$

where *x* and *y* are the *x*- and *y*-coordinates in the standard superellipse equation. We can fit the parameters of *x*_0_, *y*_0_, and θ together with the three original model parameters *a, k*, and *n*, using the optimization algorithm of Nelder and Mead (1965) [see the function “optim” in R software (R Development Core Team, 2013)]. This optimization algorithm has proven effective for estimating the parameters of a non-linear model (Shi et al., 2013). In Appendices S1-S2, we provided a MATLAB function “profile” (M-file; see Appendices S1-S1 in Supplementary Material) for extracting the planar coordinates from a tree-ring image and two R functions (i.e., “optim.sf” and “fit.sf” R-files; also see Appendices S1-S1 in Supplementary Material) for fitting the model parameters of a transformed superellipse equation (i.e., a non-standard superellipse equation). The estimated angle between the major axis and the horizontal axis for these two R functions was defined in the range of (–2π, 2π).

### Evaluation

To verify the validity of the superellipse equation and relevant R functions, we developed a “simu.sf” function (R-file; see Appendices S1-S2 in Supplementary Material) for simulating the planar coordinates based on the given model parameter values (*x*_0_ = *y*_0_ = 200, θ = π/4, *a* = 50, *k* = 0.95, and *n* = 1.9). Because the actual tree-ring shape can slightly deviate from a standard superellipse, the effects of the variation in a tree-ring boundary on the parameter estimation were considered during the simulation. Thus, the “simu.sf” function was designed to permit a variation in the radial coordinate (i.e., *r*) by setting the optional value of coefficient of variation (“CV”) in any direction. The effects of different CVs on the parameter estimation was investigated by the goodness-of-fit when CV = 4, 3, 2, 1, 0.5, and 0%.

To check how the number of the points extracted from a tree-ring image affects the parameter estimation and the model fit, data points of 200, 400, 800, 1600, 3200, and 6400 were randomly sampled from a simulated tree ring when CV = 1%. When the model parameters were given, the area encircled by a tree ring was actually fixed. For these two types of simulations, we compared the area calculated from the real model parameters and from the fitted model parameters. In general, the width of the confidence interval (CI) of a parameter estimate becomes narrower when the sample size increases. The relevant R functions to calculate the area encircled by a tree ring and the CIs of the model parameters are provided in Appendices S1 and S2.

Although these simulation methods can provide a reasonable result for evaluating model performance, the simulated data only followed a pre-defined superellipse. To evaluate whether the actual tree rings of conifers can follow the superellipse equation (i.e., whether this precondition of superellipse shape holds), it is necessary to explore the spiral growth of conifers using actual tree rings. We examined six actual cross sections collected at DBH from three tree species, including cross-sections from four white spruce (*Picea glauca* (Moench) Voss.) trees (Huang et al., 2013), one from black spruce [*Picea mariana* (Mill.) B.S.P.] (Tardif et al., 2001), and one from Douglas-fir [*Pseudotsuga menziesii* (Mirbel) Franco] (Grissino-Mayer, 1996) (see Supplementary Table 1, Supplementary Figure 2).

For each of the cross sections, we examined whether the angle (θ) between the major axis and the horizontal axis changes when tree ages over time. We defined the angle of the horizontal axis as 0, then defined the angle change due to the rotation of the major axis in an anti-clockwise direction and in a clockwise direction as a positive number and a negative number, respectively. If the angle of θ_*i*__+1_ in the (*i*+1)th year was larger than the angle of θ_*i*_ in the *i*th year, an anti-clockwise spiral growth in the increments of (θ_*i*__+1_ – θ_*i*_) was observed; otherwise, a clockwise spiral growth in the increments of (θ_*i*_ – θ_*i*__+1_) was observed. Obviously, the clockwise rotation results in a left-handed spiral grain while the anti-clockwise rotation leads to a right-handed spiral grain. As the shape of a superellipse is symmetrical around the major axis or the minor axis, the produced tree-ring shapes for the angle θ and θ ± π should be the same in theory. Assume that the real angle of a tree ring is θ_2_. Whether its estimate is ${\hat{\theta }}_{2}$ or ${\hat{\theta }}_{2}\pm \pi$ will not affect the description for tree-ring shape. However, comparison of the angles from tree rings at different ages can be negatively affected. Assume that the real angles for two adjacent tree rings are θ_1_ and θ_3_ and their corresponding estimates are ${\hat{\theta }}_{1}$ and ${\hat{\theta }}_{3}$, respectively. If the real angle θ_2_ is incorrectly estimated to be ${\hat{\theta }}_{2}$– π, tree-ring angle at the middle age can then be largely underestimated compared to the angles of the neighbors. Therefore, the R function “angle.corr” was developed to automatically correct the angles that have been overestimated or underestimated to make them rank in a normal order (see Appendices S1 and S2). This function can also detect abnormal angle estimates from bad fitting. To test whether a general trend of spiral growth within species exists, we compared the corrected angles of the four log cross sections of white spruce.

To test whether a tree ring still follows a circle equation or a pure ellipse equation rather than a superellipse equation, we further tested whether the ratios of minor to major semi-axis (values for *k*) and the powers (values for *n*) were different among the four cross sections of white spruce. If tree rings follow a circle equation, the ratios should be identical (= 1), and the powers should also be identical (= 2). If the tree rings follow a pure ellipse equation, the ratios should be smaller than 1 and the powers should be identical (= 2). Because the frequency distributions for the parameters *k* and *n* were unknown, the Kruskal-Wallis test was used to compare the difference among the four log sections (Hollander and Wolfe, 1973).

To compare the validity and complexity of the model when using the superellipse equation and the circle equation in describing tree-ring shapes, we used the Akaike Information Criterion (AIC; Burnham and Anderson, 2004), which can reflect the trade-off between the goodness-of-fit and the model complexity. Model comparisons with the AIC were performed for both the simulated and real tree rings. The simulated tree rings were produced by the superellipse equation with 0.75 < *k* < 1 and 1.7 < *n* < 2.3 for our focal species. The effect of the number of data points on a tree ring (100, 200, 400, and 800, respectively) on model performance was also checked using the AIC. Five real tree rings per cross-section were randomly chosen for white spruce, black spruce and Douglas fir, and one ring for jack pine, red pine, tamarack, and white cedar (see Table 1, Supplementary Table 2 for details).

**Table 1:** **Comparison between actual and estimated parameters under different coefficients of variation (CV) (number of data points = 1000)**.

**Parameters**	**Actual values**	**Estimates**					
		**CV = 4%**	**CV = 3%**	**CV = 2%**	**CV = 1%**	**CV = 0.5%**	**CV = 0%**
*x*_0_	200	200.24	200.13	200.01	199.96	199.99	199.92
*y*_0_	200	199.93	199.98	199.95	199.99	199.99	200.05
θ	0.7854	0.7881	0.7493	0.8042	0.7729	0.7828	0.7722
*a*	50	50.32	50.05	49.99	50.04	50.03	49.97
*k*	0.95	0.9383	0.9480	0.9517	0.9498	0.9498	0.9508
*n*	1.9	1.9105	1.9037	1.8931	1.8967	1.8997	1.9008
*R*^2^	–	0.2039	0.3101	0.4712	0.7900	0.9350	0.9927
χ^2^	–	75.8	41.3	18.6	4.7	1.2	0.1
Area (cm^2^)	7309	7328	7313	7308	7314	7315	7307

To test if the superellipse equation performs better than the traditional circle equation in estimating the basal area, we compared the goodness-of-fit (with the χ^2^ value) of the areas that were calculated using the superellipse equation and using the circle equation, when the radius is exactly equal to the major axis, the minor axis, and the average of both, respectively. The χ^2^ value was calculated by

(5) $$\begin{array}{l} \chi^{2}=\overset{q}{\underset{i=1}{\sum }}\frac{{\left(A_{i}-{Â}_{i}\right)}^{2}}{{Â}_{i}} \end{array}$$

where *A*_*i*_ and $\hat{\text{A}}$_*i*_ represent the actual basal area and the predicted basal area encircled by tree ring in year *i*, respectively; *q* represents the total number of years in a cross section. The lower the χ^2^ value, the better the fit of the model. The circle equation might often overestimate or underestimate the basal area increment in any given time of period when using the major axis or the minor axis as the radius, respectively. Therefore, average error proportion (%) in annual basal areal increment, which was defined as mean ratios of the absolute values of differences between the predicted and actual annual areal increments to the actual annual areal increments, was also calculated. An improved accuracy, as expressed by the difference of average error proportion obtained by the superellipse and the circle-3, was further calculated to assess the predicative capacity of the model.

## Results

Tree-ring shapes can be fitted by the superellipse equation with high precision. The simulations confirmed that the optimization method could produce reliable parameter estimates. The goodness-of-fit, indicated by adjusted *R*^2^ and χ^2^, declined with the increase of CV, yet the parameter estimates were still reliable even for CV = 4% (Table 1 and Supplementary Figure 3). The same pattern emerged using different numbers of data points, yet the coefficients of determination remained almost unchanged (Table 2 and Supplementary Figure 4). A narrower 95% CI for any parameters was found when the number of data points increased. The 95% CI of the parameter θ only had an absolute difference of 0.03 (< 5% of the real value) for a sample size ≥ 800.

**Table 2:** **Comparison between actual and estimated parameters under different numbers of data points (CV = 1%)**.

**Parameter**	**Real value**	**Estimate (with 95% CI)**		
		**Size = 200**	**Size = 400**	**Size = 800**
*x*_0_	200	199.93 (199.82,200.02)	200.01 (199.94,200.08)	199.98 (199.94,200.02)
*y*_0_	200	200.08 (199.97,200.16)	200.04 (199.97,200.11)	200 (199.95,200.04)
θ	0.7854	0.7935 (0.7619,0.8296)	0.7921 (0.7705,0.8166)	0.7803 (0.7647,0.7938)
*a*	50	50.03 (49.85,50.15)	50.14 (50.04,50.26)	49.99 (49.92,50.06)
*k*	0.95	0.9479 (0.9449,0.9519)	0.9476 (0.9446,0.9505)	0.95 (0.948,0.9515)
*n*	1.9	1.9134 (1.895,1.9388)	1.8898 (1.8733,1.9034)	1.9028 (1.8951,1.9125)
*R*^2^	–	0.7937 (0.7501,0.841)	0.7884 (0.7583,0.8168)	0.7888 (0.7665,0.8135)
χ^2^	–	0.96 (0.77,1.11)	1.97 (1.71,2.2)	3.75 (3.3,4.09)
Area (cm^2^)	7309	7324 (7301,7341)	7316 (7301,7331)	7312 (7303,7320)
**Parameter**	**Real value**	**Estimate (with 95% CI)**		
		**Size = 1600**	**Size = 3200**	**Size = 6400**
*x*_0_	200	199.99 (199.96,200.02)	200 (199.96,200.13)	199.99 (199.97,200.02)
*y*_0_	200	200 (199.97,200.04)	199.98 (199.96,200.13)	200 (199.98,200.04)
θ	0.7854	0.7912 (0.7812,0.8027)	0.7844 (0.7711,0.8017)	0.7891 (0.7817,0.7967)
*a*	50	50.04 (49.97,50.09)	49.99 (49.89,50.24)	50.01 (49.91,50.01)
*k*	0.95	0.9491 (0.9477,0.9506)	0.9493 (0.945,0.95)	0.9497 (0.9491,0.9509)
*n*	1.9	1.8992 (1.8929,1.906)	1.9052 (1.8855,1.9205)	1.9017 (1.9002,1.913)
*R*^2^	–	0.79 (0.7736,0.8049)	0.7764 (0.7569,0.7863)	0.7815 (0.7725,0.7887)
χ^2^	–	7.64 (7.08,8.15)	16.64 (16.02,17.97)	31.67 (30.7,32.86)
Area (cm^2^)	7309	7311 (7303,7315)	7308 (7296,7328)	7311 (7302,7314)

**Figure 3:**
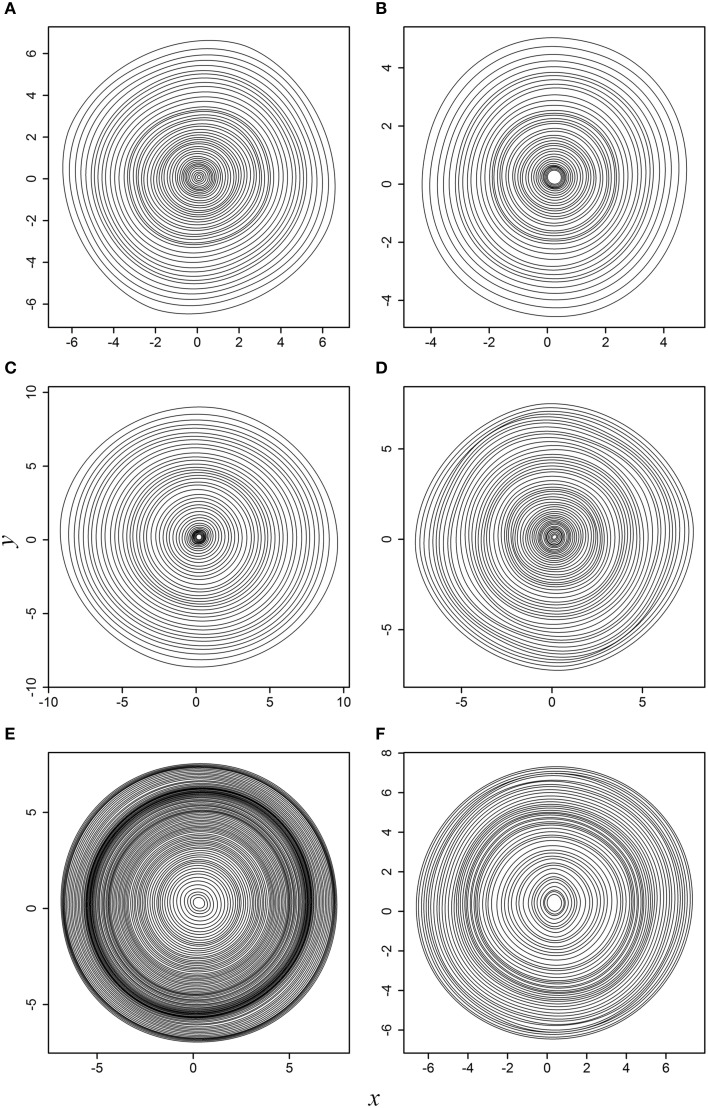
**Fitted results to the six actual tree cross sections: (A) WS-1; (B) WS-2; (C) WS-3; (D) WS-4; (E) black spruce; (F) Douglas-fir**. This figure corresponds to Supplementary Figure 2.

When actual tree-ring samples were used, the superellipse model was able to fit the planar coordinates. Figure 3 displays the fitted results for the six actual tree cross sections. For any tree cross section, each of the predicted tree rings is symmetrical around its major axis; however, such symmetry is difficult to be observed intuitively when all tree rings are superposed together due to the angle rotation.

**Figure 4:**
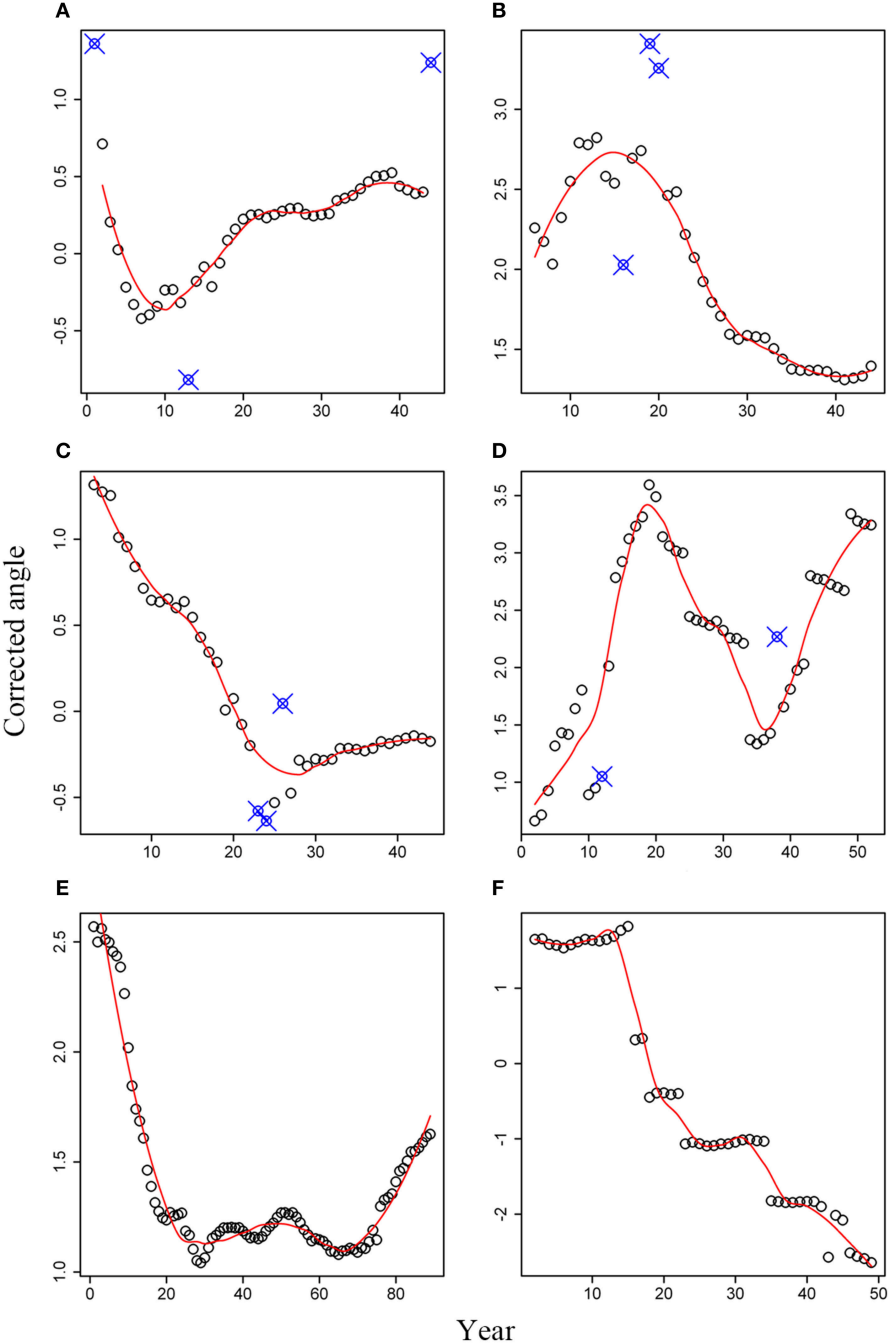
**Corrected angles for six tree cross sections: (A) WS-1; (B) WS-2; (C) WS-3; (D) WS-4; (E) black spruce; (F) Douglas-fir**. Small open circles represent the corrected angles; Solid line represents the predicted values based on the local regression method; Small open circles with signs of “X” represent abnormal data that were not used.

For white spruce (Figures 4A–D), a general pattern of angle rotation was not found, even for WS-1 and WS-2 collected from the same site but exhibited different trends in the angle change (Figures 4A,B). As shown in Figure 4A, an obvious low point appeared in the 10th ring for this white spruce. Therefore, tree rings at age ≤ 10 years rotated in a clockwise direction, resulting in a left-hand spiral grain over the longitudinal axis. Tree rings at age > 10 years rotated in a reverse clockwise direction, leading to a right-hand spiral grain over the longitudinal axis. In contrast to WS-1, white spruce WS-2 showed an inverse trend, with a peak point at age of 15 years (Figure 4B). The reverse clockwise rotation was observed at age ≤ 15 and the clockwise rotation was found at age > 15. Correspondingly, the right-hand and left-hand spiral grain over the longitudinal axis was expected, respectively. The angle change trend in WS-3 was more or less similar to that of WS-1, but its lowest point appeared in the 25th ring (Figure 4C). Interestingly, white spruce sample WS-4 exhibited a completely different trend from those of the first three white spruce trees. As shown in Figure 4D, a peak point occurred in the 19th year and a lowest point appeared in the 36th year. Therefore, tree rings first rotated in an anti-clockwise direction at age ≤ 19, then rotated in a clockwise direction at 19 < age ≤ 36, and finally rotated in an anti-clockwise direction at age > 36 again.

Tree-ring angles of black spruce kept a continuous decrease during the first 30 years, indicating a clockwise rotation (Figure 4E). However, the angle changes were not very substantial from the 30th year to the 70th year. Afterwards, tree-ring angles began to become larger and larger, which means an anti-clockwise rotation. Compared to tree species mentioned above, tree-ring angles for Douglas-fir have its distinct rotation, which was characterized by several plateaus and an overall decreasing trend over time (Figure 4F).

**Figure 5:**
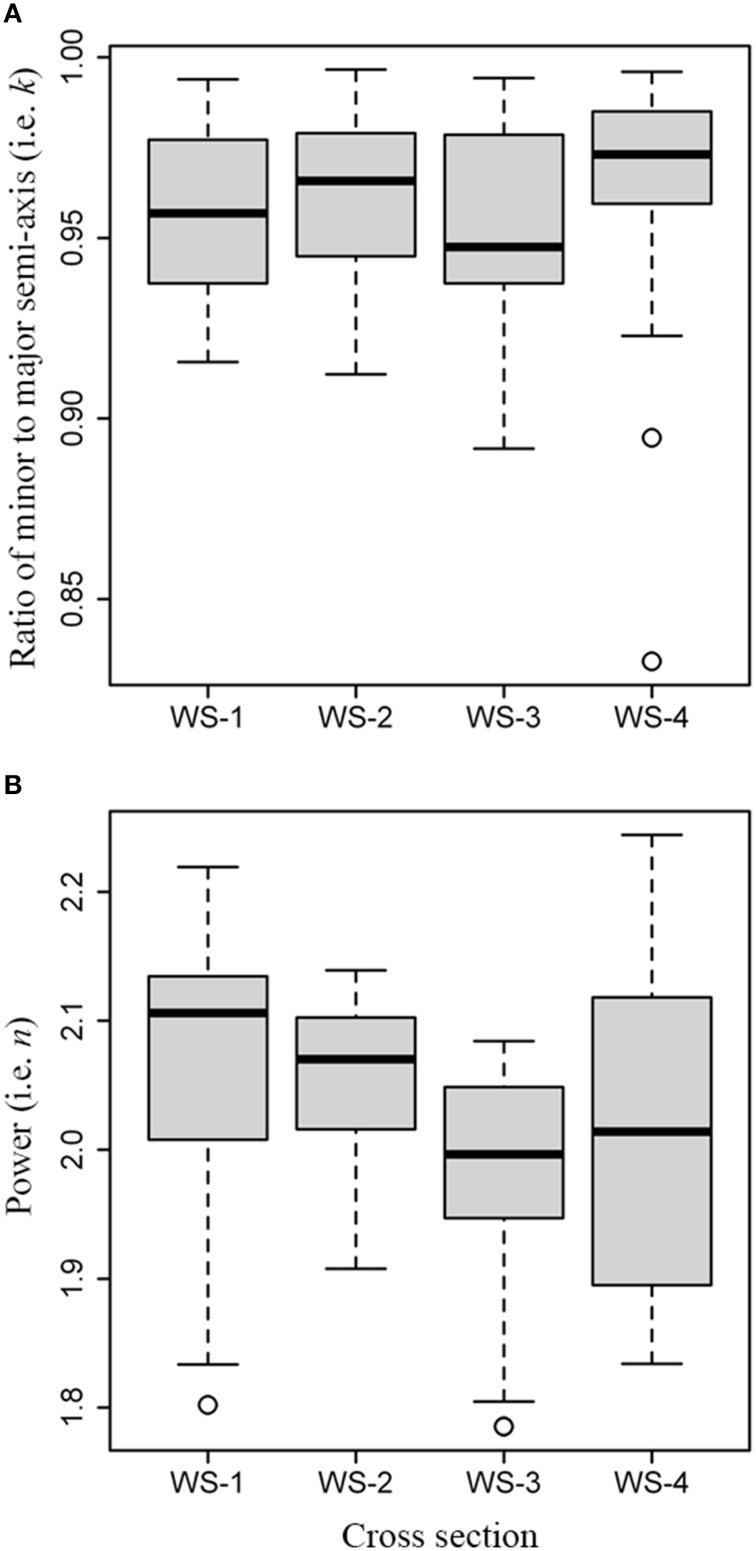
**Comparison of values for ***k*** and ***n*** among the cross sections from white spruce: (A) boxplot for values of ***k***; (B) boxplot for values for ***n*****.

The Kruskal-Wallis test showed a significant difference in the ratios of minor to major semi-axis (χ^2^ = 12.1, *df* = 3, *P* = 0.007; Figure 5A), and a significant difference in the powers *n* (χ^2^ = 23.2, *df* = 3, *P* < 0.01; Figure 5B) among the four samples of white spruce. The pairwise test for the median of *k* showed insignificant difference between any pair of WS-1, WS-2, and WS-3 (*P* > 0.05), except for WS-4 which showed significant differences with WS-1 and with WS-3 (*P* < 0.05), while significant difference in the median of *n* between any pair of WS-1, WS-2, and WS-3 (*P* < 0.05) was found, except for WS-4 which showed significant difference with WS-1 (*P* < 0.05) only. Tree-ring shape is not a standard ellipse because the values for *n* from the first two cross sections are higher than 2 (Figure 5B). It also showed that the ratios and powers in the superellipse equation can be different even for the four samples from the same species.

**Figure 6:**
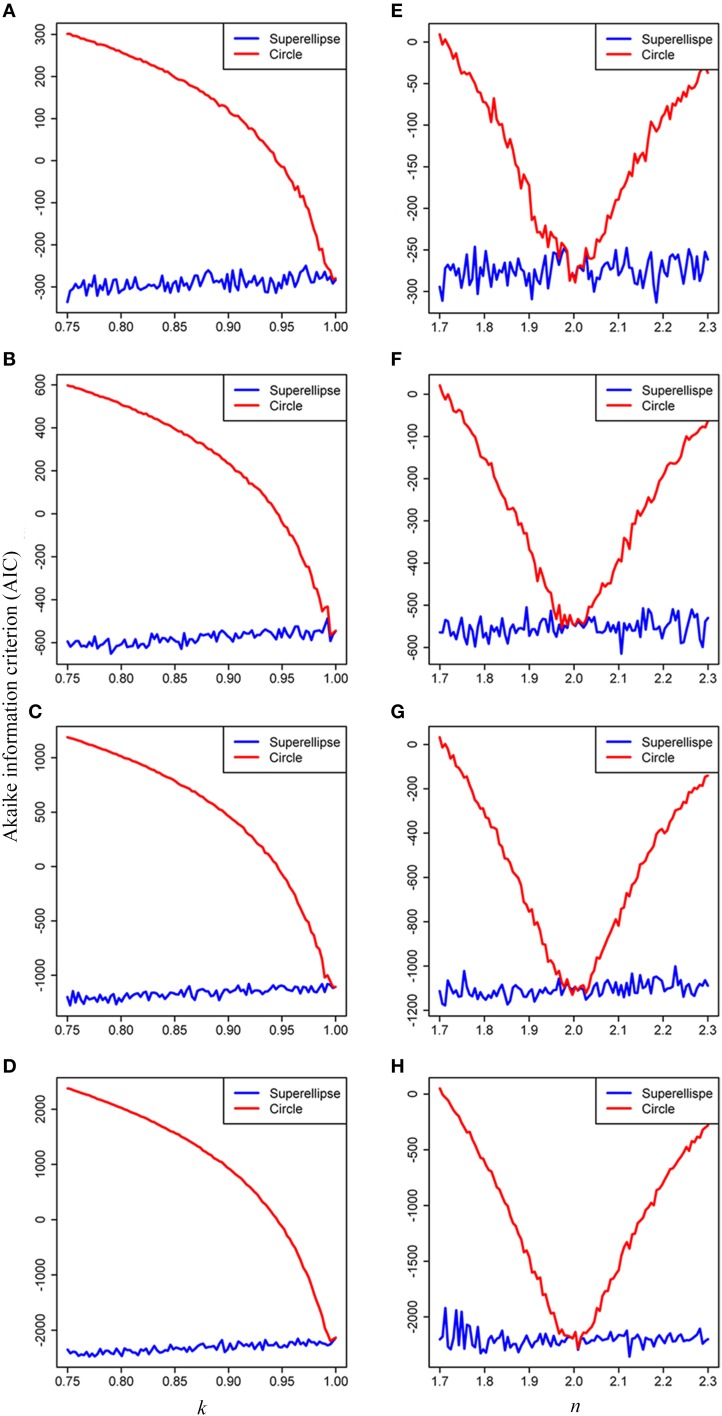
**Effects of the parameters ***k*** and ***n*** on the Akaike Information Criterion (AIC) when simulating tree rings by the superellipse and circle equations with 100 (A,E), 200 (B,F), 400 (C,G), and 800 (D,H) data points, respectively**. The simulated tree rings were produced by the superellipse with *x*_0_ = *y*_0_ = 200, *a* = 50, *n* = 2, and 0.75 < *k* < 1 for panels **(A–D)**, with *x*_0_ = *y*_0_ = 200, *a* = 50, *k* = 1, and 1.7 < *n* < 2.3 for panels **(E–H)**. We permitted 5% coefficient of variation in the distances of data points on a simulated tree ring from the corresponding pole.

The results of model comparison using the AIC showed that the superellipse equation performed better than the circle equation in describing the real tree-ring shapes of conifers. The AIC values obtained from the superellipse equation were lower than those obtained from the circle equation when *k* < 1, *n* < 2 and *n* > 2 (Figure 6). It implicates that the superellipse equation is generally better than the circle equation in describing the simulated tree-ring shapes except when the tree-ring shape is perfectly round. The results also showed a decline of the AIC score with the increase of data points on a simulated tree ring. The same conclusion of the superellipse equation superior to the circle equation was also drawn from using the AICs for real tree rings (see Supplementary Table 2) as the estimated *k* is usually smaller than 1 and *n* unequal to 2 for any real tree rings.

In addition, the results showed that the superellipse equation had the lowest χ^2^ values which mean the best goodness-of-fit compared to the other three calculations using the circle equation, as shown in Table 3. Average error proportion calculated by the superellipse was much lower than that calculated by the circle. The results of improved predicative accuracy between the superellipse and the circle-3 showed that the superellipse generally had higher accuracy than the circle in predicting the basal area increment, ranging from 2.31 to 12.57% for our focal species (Table 3).

**Table 3:** **Comparison of the goodness-of-fit (χ^2^) among the predicted basal areas by different equations**.

**Sample**	**Superellipse^a^(×10^−4^)**	**Circle-1^b^**	**Circle-2^c^**	**Circle-3^d^**	**AEP_S (%)**	**AEP_C3 (%)**	**Accuracy (%)**
WS-1	<0.1	2.06	5.57	1.09	0.15	7.18	7.03
WS-2	1.5	1.00	3.21	0.19	0.14	4.06	3.92
WS-3	5.2	7.92	1.55	0.25	0.17	3.38	3.21
WS-4	5.2	4.82	2.88	2.31	0.25	12.82	12.57
Black spruce	3.8	5.24	2.98	0.41	0.57	4.18	3.61
Douglas fir	2.1	3.36	1.97	1.18	0.24	12.55	2.31

a *Value represents the χ^2^ value between the actual areas at different ages and the predicted areas using the superellipse equation*.

b *Value represents the χ^2^ value using the circle equation when the radius equals the major semi-axis (Circle-1)*.

c *Value represents the χ^2^ value using the circle equation when the radius equals the minor semi-axis (Circle-2)*.

d *Value represents the χ^2^ value using the circle equation when the radius equals the mean of both the major semi-axis and the minor semi-axis. AEP indicates average error proportion (%) in predicted basal areal increment between two adjacent rings by the superellipse (AEP_S) and the circle-3 (AEP_C3). Because the Circle-3 can get the lowest χ^2^ value among the three circle equations, we only used the Circle-3 here. WS1 to WS4 were from white spruce. An improved accuracy (Accuracy) between the superellipse and the circle-3 was calculated as the difference between AEP_C3 and AEP-S*.

## Discussion

### Tree-ring shape and improved estimates of the basal area

Our modeling results showed that tree-ring shape can be best fitted by the superellipse equation with high precision. This suggests that tree rings do not follow either a circle equation or a pure ellipse equation, but somewhere in-between, i.e., a superellipse equation. Theoretically, tree-ring shape should be a circle under genetic influences only because cambium cell differentiation of a healthy tree is assumed to be at the same rate along the circumference during the growing season (Lupi et al., 2014). However, due to the influences of external factors such as environmental stresses (e.g., light availability, water stress) and biogeophysical factors (e.g., position, slope), trees have to physiologically adjust the rate of cambium cell differentiation along the circumference during the growing seasons to survive in or adapt to the local environmental conditions. Consequently, a “superelliptical” tree ring is produced, as widely observed in terrestrial forest ecosystems. Local conditions in the cambium that influence wood formation at any given instant are believed to be unique because the immediate environment of a cambial initial (weather and nutrient factors, growth regulators, physical stresses) varies continuously over time (Downes et al., 2009). A recent micro-sampling based study investigated the process of cambium cell differentiation of black spruce along the circumference during the growing season, and found that the onset of xylogenesis along the circumference varies within an individual tree (Lupi et al., 2014). More earlywood cells (corresponding to a wider annual ring) were often found in a warm year than a cold year (Rossi et al., 2007; Deslauriers et al., 2008; Huang et al., 2011), suggesting that xylem cell number is mainly determined by environmental factors such as temperature. The importance of various drivers of xylogenesis may shift from factors mainly varying at the site level (e.g., climate) at the beginning of the growing season to factors varying at the individual tree level (e.g., possibly genetic variability) at the end of the growing season (Lupi et al., 2014).

The results of comparison of goodness-of-fit (χ^2^) among the areas predicted by different equations showed that tree-ring shape can be best fitted by the superellipse equation compared to by the circle equation, i.e., by the major semi-axis or the minor semi-axis or the average from both. Basal area is one of the basic parameters in forestry and forest ecology and has been widely used in estimates of forest growth and productivity as well as carbon storage and equilibrium (Phillips et al., 1998; Ma et al., 2012). Our results might indicate that traditional circle-based estimates of the basal area might be more or less over- or under-estimated in practice because traditional calculations of the basal area were, directly or indirectly, based on the DBH. DBH values measured in practice may range from the length of the major axis to that of the minor axis due to its feature of spiral growth of the cross-section over age (West, 2009; Torres and Lovett, 2013). The improved accuracy obtained by the superellipse than the other commonly used circle-based approaches might also indicate an improved estimate of the basal area through the superellipse fit. This may contribute to a better understanding of forest growth and productivity, as well as carbon balance and dynamics in terrestrial forest ecosystems given that forests cover 31% of the world's land surface (FAO, 2010).

All evidence together suggests that tree-ring geometric shape can be better depicted by the superellipse than the circle commonly used in practice. Although our study focused on coniferous species only due to its clear annual ring pattern, this geometric shape of tree rings is believed to be universal and common in deciduous species as well.

### Spiral growth on the cross section

Our study first quantitatively confirmed that radial growth follows a spiral growth pattern over time despite that the angle change between successive years being much smaller than reported previously in spiral grain-based studies, such as 30–50° in ponderosa pines (*Pinus ponderosa*) (Leelavanichkul and Cherkaev, 2004; Wing et al., 2014). It is widely accepted that spiral grain originates from the cambium and spiral grain formation can be coupled to cell divisions taking place in the cambial region (Harris, 1989; Eklund and Säll, 2000). Therefore, spiral growth over the cross section might be closely related to spiral grain over the longitudinal axis although this potential relationship needs to be further quantified. Previous studies have attempted to explore the spiral grain angles over time along the longitudinal axis (Danborg, 1994; Gjerdrum et al., 2002; Watt et al., 2013), but the potential relationship between the rotation angles of tree rings on the cross-section over time and the grain angles along the longitudinal axis over time was not quantified. Although spiral grain pattern over the longitudinal axis for many tree species is less visible or even undetected due to a minor change in the shifting angle, we conclude that spiral growth over the cross section might be common in many tree species, including both coniferous and deciduous species.

Previous studies often claim that coniferous trees shift the direction of spiral grain pattern from LR to RL or vice versa, but rarely clearly confirm the exact year when this shift occurred (Kubler, 1991). One or two extreme points observed in the angle change over time in our study might indicate the exact or critical year or years when the direction of spiral grain over the longitudinal axis shifted from LR to RL or from RL to LR. However, the potential mathematical link between the spiral growth over the cross section and spiral grain over the longitudinal axis has not been elucidated yet and thus merits further investigation. In addition, the extreme points found in early years or late years in our study also suggest that the direction shift in spiral grain, either LR to RL or RL to LR pattern, may occur in both young and old stages of tree growth. These findings are counter to the results from previous studies that found a LR pattern normally formed in early stages of growth but gradually shifted to a RL pattern in the older stages of growth for coniferous trees in the northern hemisphere. Further, our results counter the suggestion that two shifts in spiral grain pattern are rarely observed (Skatter and Kucera, 1998). These differences might be partly attributed to different perspectives of investigation, i.e., longitudinal axis vs. cross section. In addition, spiral grain over the longitudinal axis or spiral growth over the cross section is a time-dependent growth phenomenon and is difficult to monitor over time. Most of the previous studies were based on field observations with a quantitative analysis over time lacking. Consequently, the complexity of the mechanisms behind spiral grain over the longitudinal axis might be underestimated.

Fluctuations in the corrected angles of the radial section over time differed within a site and across sites and species. This finding indicates that spiral growth on the radial section is determined less by population and species genetics, but more by macro- and micro-environmental factors. Our findings are contrary to some of the previous studies that claimed that spiral grain pattern in conifers is strongly genetic (Kubler, 1991; Skatter and Kucera, 1998) such as Sitka spruce [*Picea sitchensis* (Bong.) Carr.] (Hansen and Roulund, 1997) and loblolly pine (*Pinus taeda* L.) (Zobel et al., 1968). The results from our study are in general agreement with other studies that claimed that spiral grain is strongly affected by environmental factors such as wind (Koizumi et al., 2007). Overall, various hypothetical reasons for spiral grain have been proposed, such as the Earth rotation, optimal structure for even distribution of sap between the roots and the crown, wind torque, and the relief of growth stresses in the cambial zone (Wing et al., 2014). Still, a consensus has not been reached. From the perspectives of ecology and evolution, we infer that spiral growth over the cross section, which is closely related to spiral grain over the longitudinal axis, is controlled by both genetic and environmental factors, but the importance of both factors in affecting spiral growth or spiral grain might be shifting over time, i.e., site and species- specific, even individually.

### Model advantages and limitations

We have justified that the superellipse equation is superior to the traditional circle equation in modeling tree-ring boundary as according to the AIC. We are confident that the superellipse equation not only fits tree-ring shapes of coniferous species, but also can be widely applied in fitting tree-ring shape of many tree species. Supplementary Figures 5, 6 exhibited the application of the superellipse equation on additional four species of conifers. The most important advantage is that estimates of forest growth and productivity as well as carbon storage can be improved when the superellipse equation is employed in the future, in contrast to the ill-fitting of tree-ring shapes by the circle equation.

The standard superellipse includes three parameters (i.e., *a, k*, and *n*) whereas the circle equation only has a single parameter (radius), reflecting a more complex structure of the former than the latter. In general, for model selection, the adjusted coefficient of determination (*R*^2^_*adj*_), AIC, corrected AIC, Bayesian information criterion (BIC), deviance information criterion (DIC), and residual information criterion (RIC) are better than the RSS, *R*^2^, and χ^2^ given the trade-off between the goodness-of-fit of the model and the complexity of the model (see Shi and Ge, 2010 and references therein). In practice, forest growth and productivity as well as carbon storage might have often been under- or over-estimated due to the ill-fitting of tree-ring shapes by the circle equation. To better estimate forest productivity and carbon dynamics in forest ecosystems, investigators are usually concerned less about the model structural complexity, but more on the goodness-of-fit of the model. That means, the better the fitting is, the better a model would be. Given that the original input parameters of our model are obtained from the geometric shape of tree-ring boundary rather than ring-width measurements from a single core or two cores or cross sections commonly collected at DBH from the field, our model is convenient to be applied in practice if the cross-section of trees can be collected in the field and then the parameters of tree-ring shape can be obtained using our method (see Appendices S1–S2 in Supplementary Material). Otherwise currently it is less convenient to obtain these parameters due to a lack of such a tool to automatically measure tree-ring shape in the field, which however deserves to be further invented for the practitioners.

Although our study showed that the suprellipse equation could fit the tree-ring shapes of conifers very well, it is only a descriptive model. A better model for describing tree-ring shape and growth should be based on the dynamics of growth for trees, especially necessarily considering the biophysical mechanism of tree stem formation accompanied with growth stresses (Archer, 1989). Although no convincing evidence has demonstrated that the macrocosmic spiral growth in tree trunk could be related to microcosmic microfibril angle in fiber (Barnett and Bonham, 2004; Jordan et al., 2007), there might be a certain relationship between them. Thus, a mechanical model that could link the effects of growth stresses on the microfibril angle in fiber to the spiral growth in conifers merits further investigation.

### Author contributions

PS and JH designed the study. PS analyzed the data. JH and PS wrote the paper. All the coauthors discussed and commented the paper.

## Conflict of interest statement

The authors declare that the research was conducted in the absence of any commercial or financial relationships that could be construed as a potential conflict of interest.
