# Supplementary material for: Capturing spiral radial growth of conifers using the superellipse to model tree-ring geometric shape
Source: Front Plant Sci. 2015 Oct 15;6:856. doi: 10.3389/fpls.2015.00856 (PMC4606055; doi:10.3389/fpls.2015.00856)
Supplement: Supplementary file 1 [file DataSheet1.PDF]

Supplementary Material

Capturing spiral radial growth of conifers using the superellipse to model tree-ring geometric shape

Pei-Jian Shi[1,2#], Jian-Guo Huang[2,#*], Cang Hui[3,4], Henri D. Grissino-Mayer[5], Jacques Tardif[6], Li-Hong Zhai[2], Fu-Sheng Wang[1], Bai-Lian Li[7]

[1] Co-Innovation Centre for Sustainable Forestry in Southern China, Bamboo Research Institute, Nanjing Forestry University, Nanjing, China
[2] Key Laboratory of Vegetation Restoration and Management of Degraded Ecosystems, Provincial Key Laboratory of Applied Botany, South China Botanical Garden, Chinese Academy of Sciences, Guangzhou, China
[3] Centre for Invasion Biology, Department of Mathematical Sciences, Stellenbosch University, Matieland, South Africa
[4] Mathematical and Physical Biosciences, African Institute for Mathematical Sciences, Cape Town, South Africa
[5] Department of Geography, The University of Tennessee, Knoxville, TN, USA
[6] Centre for Forest Interdisciplinary Research (C-FIR), University of Winnipeg, Winnipeg, MB, Canada
[7] Ecological Complexity and Modelling Laboratory, Department of Botany and Plant Sciences, University of California, Riverside, CA, USA

[#] Equal contribution.

*** Correspondence:** Jian-Guo Huang, Key Laboratory of Vegetation Restoration and Management of Degraded Ecosystems, Provincial Key Laboratory of Applied Botany, South China Botanical Garden, Chinese Academy of Sciences,723 Xingke Road, Guangzhou, 510650, China.
huangjg@scbg.ac.cn

1. Supplementary Data

Appendix S1. MATLAB and R functions developed.

Appendix S2. The usage of functions developed in the present study.

2. Supplementary Figures and Tables

Supplementary figures and tables are complementary materials of main file.

2.1. Supplementary Tables

Supplementary Table 1 | Information of the cross sections used in the study

Species	Latin name	Latitude	Longitude	Location	First ring	Last ring
White spruce (WS-1)	*Picea glauca*	55.03	111.68	Alberta, Canada	1964	2007
WS-2	*Picea glauca*	55.03	111.68	Alberta, Canada	1964	2007
WS-3	*Picea glauca*	55.07	111.81	Alberta, Canada	1964	2007
WS-4	*Picea glauca*	54.83	111.84	Alberta, Canada	1957	2007
Black spruce	*Picea mariana*	52.68	101.37	Manitoba, Canada	1919	2008
Douglas fir	*Pseudotsuga menziesii*	35.17	108.32	New Mexico, USA	1951	1998
Jack pine	*Pinus banksiana*	50.68	95.35	Manitoba, Canada	1942	2007
Red pine	*Pinus resinosa*	47.23	95.2	Minnesota, USA	1982	2001
Tamarack	*Larix laricina*	53.20	100.60	Manitoba, Canada	1850	2004
White cedar	*Thuja occidentalis*	52.90	99.20	Manitoba, Canada	1828	2005

frontiers

Supplementary Table 2 | Comparison between the superellipse and circle equations in describing the real tree-ring shapes based on the Akaike information criterion (AIC)

Species	Ring location	Equation	*N*	RSS	*K*	*L*	AIC
WS-1 (White spruce)	Inner 6	Superellipse	1174	0.0166	4	6555	-13101
		Circle	1174	0.0455	2	5963	-11922
	Inner 7	Superellipse	2691	0.0797	4	14030	-28052
		Circle	2691	0.2695	2	12391	-24777
	Inner 25	Superellipse	3554	1.8248	4	13460	-26911
		Circle	3554	25.7666	2	8755	-17506
	Inner 31	Superellipse	3127	5.1315	4	10026	-20044
		Circle	3127	32.6964	2	7130	-14257
	Inner 39	Superellipse	4546	18.9100	4	12461	-24915
		Circle	4546	31.0767	2	11332	-22660
WS-2	Inner 2	Superellipse	380	0.0007	4	2500	-4993
		Circle	380	0.0040	2	2178	-4352
	Inner 6	Superellipse	1067	0.0139	4	5999	-11991
		Circle	1067	0.0234	2	5723	-11441
	Inner 10	Superellipse	1702	0.0514	4	8858	-17707
		Circle	1702	0.1319	2	8055	-16106
	Inner 31	Superellipse	2062	2.8318	4	6795	-13582
		Circle	2062	4.7653	2	6258	-12512
	Inner 37	Superellipse	2192	2.0452	4	7647	-15286
		Circle	2192	16.8910	2	5333	-10662
WS-3	Inner 3	Superellipse	504	0.0028	4	3045	-6082
		Circle	504	0.0033	2	3005	-6006
	Inner 7	Superellipse	1061	0.0548	4	5236	-10465
		Circle	1061	0.1375	2	4748	-9493
	Inner 11	Superellipse	1863	0.7404	4	7294	-14580
		Circle	1863	2.0772	2	6333	-12662
	Inner 21	Superellipse	2367	1.1240	4	9057	-18105
		Circle	2367	1.4947	2	8719	-17435
	Inner 25	Superellipse	1663	1.8591	4	5651	-11294
		Circle	1663	2.3423	2	5459	-10914
WS-4	Inner 4	Superellipse	1525	0.0186	4	8628	-17247
		Circle	1525	0.0964	2	7373	-14741
	Inner 12	Superellipse	4088	0.2128	4	20161	-40313
		Circle	4088	0.2388	2	19925	-39846
	Inner 18	Superellipse	3521	1.0629	4	14270	-28531
		Circle	3521	2.9863	2	12451	-24898
	Inner 26	Superellipse	2414	5.0600	4	7444	-14881

		Circle	2414	7.7942	2	6923	-13842
	Inner 36	Superellipse	2999	36.7027	4	6603	-13197
		Circle	2999	40.2196	2	6465	-12927
Black spruce	Inner 2	Superellipse	520	0.0265	4	2570	-5132
		Circle	520	0.0886	2	2256	-4508
	Inner 7	Superellipse	2086	0.6417	4	8434	-16861
		Circle	2086	1.9225	2	7290	-14576
	Inner 11	Superellipse	3187	0.7290	4	13358	-26708
		Circle	3187	1.3178	2	12415	-24826
	Inner 23	Superellipse	2726	3.9439	4	8912	-17816
		Circle	2726	5.8726	2	8369	-16734
	Inner 40	Superellipse	2936	3.2755	4	9980	-19952
		Circle	2936	9.0339	2	8491	-16977
Douglas fir	Inner 13	Superellipse	4139	1.2949	4	16700	-33393
		Circle	4139	3.2689	2	14784	-29564
	Inner 19	Superellipse	6160	5.0615	4	21881	-43754
		Circle	6160	8.8240	2	20169	-40334
	Inner 22	Superellipse	7065	9.0227	4	23538	-47067
		Circle	7065	15.7596	2	21568	-43131
	Inner 39	Superellipse	5306	17.7247	4	15126	-30245
		Circle	5306	32.4108	2	13525	-27047
	Inner 46	Superellipse	5985	20.9098	4	16928	-33848
		Circle	5985	39.3160	2	15038	-30073
Jack pine	-	Superellipse	3653	14.5420	4	10094	-20179
		Circle	3653	26.4860	2	8999	-17993
Red pine	-	Superellipse	3463	0.5647	4	15101	-30194
		Circle	3463	1.1470	2	13874	-27744
Tamarack	-	Superellipse	3111	0.5984	4	13309	-26611
		Circle	3111	2.4432	2	11121	-22238
White cedar	-	Superellipse	4844	75.0160	4	10094	-20181
		Circle	4844	313.1817	2	6633	-13262

Here, “WS” represents the white spruce; “Inner *i*” represents the *i*-th tree ring from the pith on a cross section; *N* represents the number of data points on a scanned tree ring; RSS represents the residual sum of squares between the observed and predicted distances of data points on a tree ring from the pole (or the centre for a circle); *K* denotes the number of model parameter including the error; *L* represents the maximized log-likelihood value. The detailed calculation method of AIC could be found in Shi and Ge (2010). For the last four species, each species one tree ring was chosen, which was marked in red on the corresponding cross section (see Supplementary Figure 5 for details). The detailed information of species and cross sections can be found in the text.

2.2. Supplementary Figures

Supplementary Figure 1. Spiral grain of dragon juniper, *Sabina chinensis* (L.) Ant. cv. Kaizuca. This photo was taken on campus of Nanjing Forestry University, Nanjing City, Jiangsu province, China (32°4'49" N, 118°48'32" E), in the late January, 2015.

Supplementary Figure 2. Six actual tree cross sections: (a) WS-1 (14.48 cm × 14.49 cm); (b) WS-2 (10.22 cm × 10.86 cm); (c) WS-3 (20.21 cm × 19.02 cm); (d) WS-4 (16.27 cm × 15.73 cm); (e) black spruce (16.97 cm × 17.42 cm); (f) Douglas-fir (16.26 cm × 16.32 cm). Dimensions represent the width and height of original photo edges. "WS" = white spruce.

Supplementary Figure 3. Comparison of simulated tree rings (gray solid lines) with predicted tree rings (red solid lines) under different values for CV: (a) 0%; (b) 0.5%; (c) 1%; (d) 2%; (e) 3%; (f) 4%. Here, $x_0 = y_0 = 200$, $\theta = \pi/4$, $a = 50$, $k = 0.95$, and $n = 1.9$. The number data points in every tree ring is 1000. The dark dashed line represents the direction of the major axis.

Supplementary Figure 4. Comparison of simulated tree rings (gray solid lines) with predicted tree rings (red solid lines) under different numbers of data points: (a) 200; (b) 400; (c) 800; (d) 1600; (e) 3200; (f) 6400. Here, $x_0 = y_0 = 200$, $\theta = \pi/4$, $a = 50$, $k = 0.95$, and $n = 1.9$. The CV in radial coordinate (i.e. r) for every tree ring is 1%. The dark dashed line represents the direction of the major axis.

Supplementary Figure 5. Additional tree cross sections from four species of conifers: (a) jack pine (*Pinus banksiana* Lamb.); (b) red pine (*Pinus resinosa* Aiton); (c) tamarack (*Larix laricina* (Du Roi) K.Koch); (d) white cedar (*Thuja occidentalis* L.). The red line was randomly chosen for carrying out a fitting.

Supplementary Figure 6. Fitted results for selected annual rings from samples shown in the preceding supplementary figure. Gray lines are those observed annual rings randomly chosen from the log cross sections, while the red lines are predicted annual rings. All estimates of k are significantly less than 1 by observing that the 95% confidence interval of $(k - 1)$ whether includes zero or is less than zero (using the bootstrap percentile method).
