# Supplementary figures and images for: Capturing spiral radial growth of conifers using the superellipse to model tree-ring geometric shape
Source: Front Plant Sci. 2015 Oct 15;6:856. doi: 10.3389/fpls.2015.00856 (PMC4606055; doi:10.3389/fpls.2015.00856)

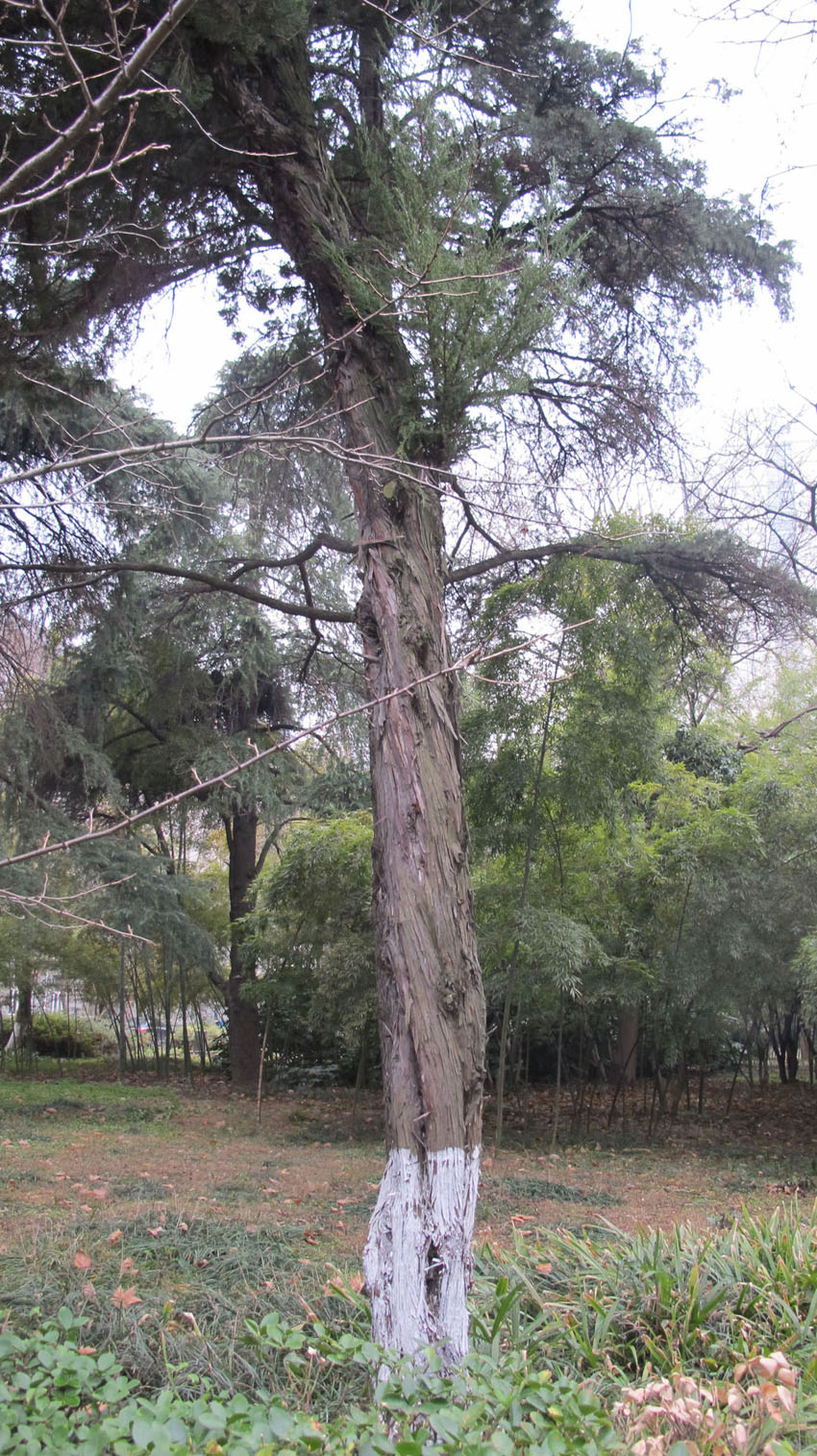

Supplement: Supplementary file 3 [file Image1.JPEG]

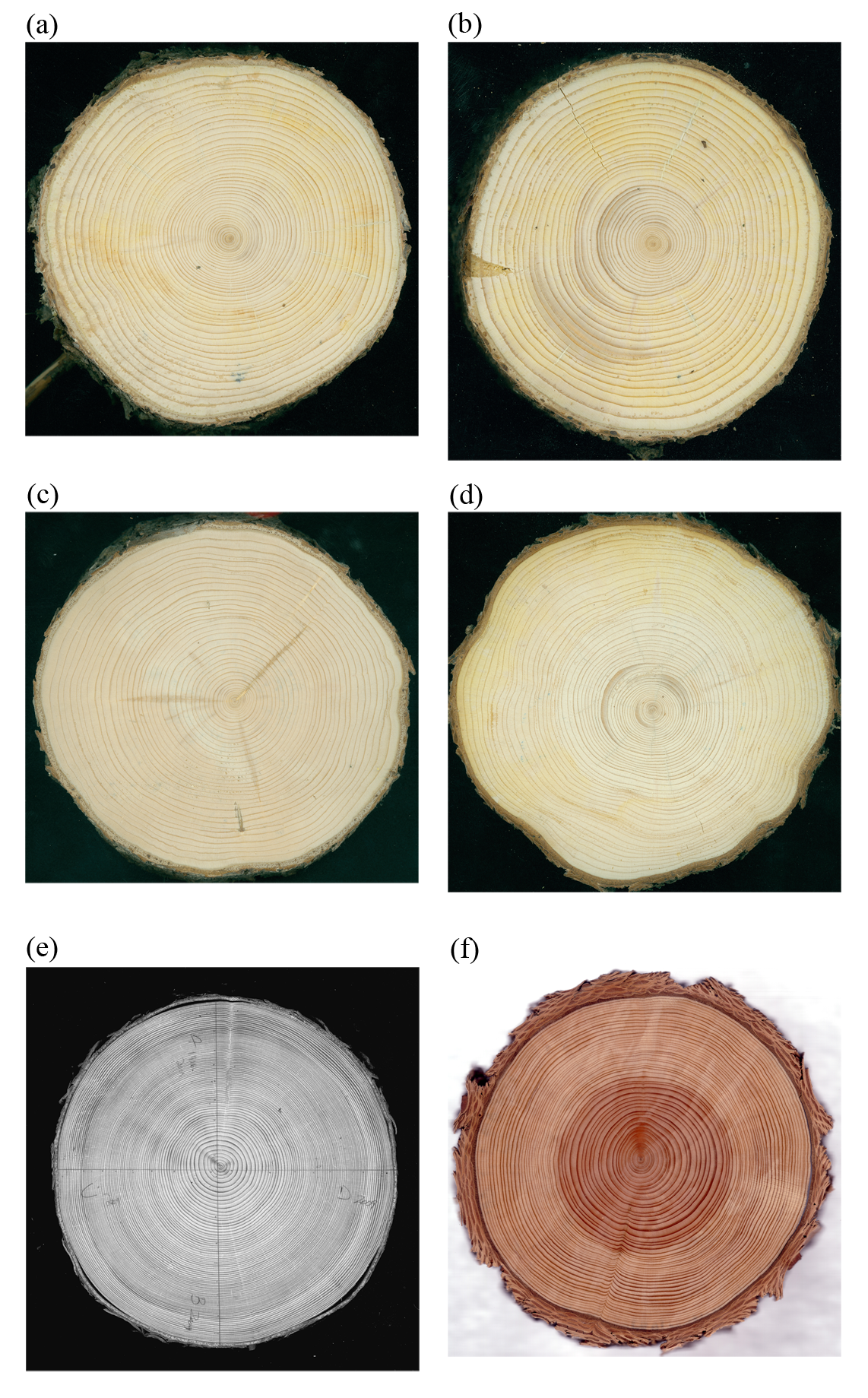

Supplement: Supplementary file 4 [file Image2.TIF]

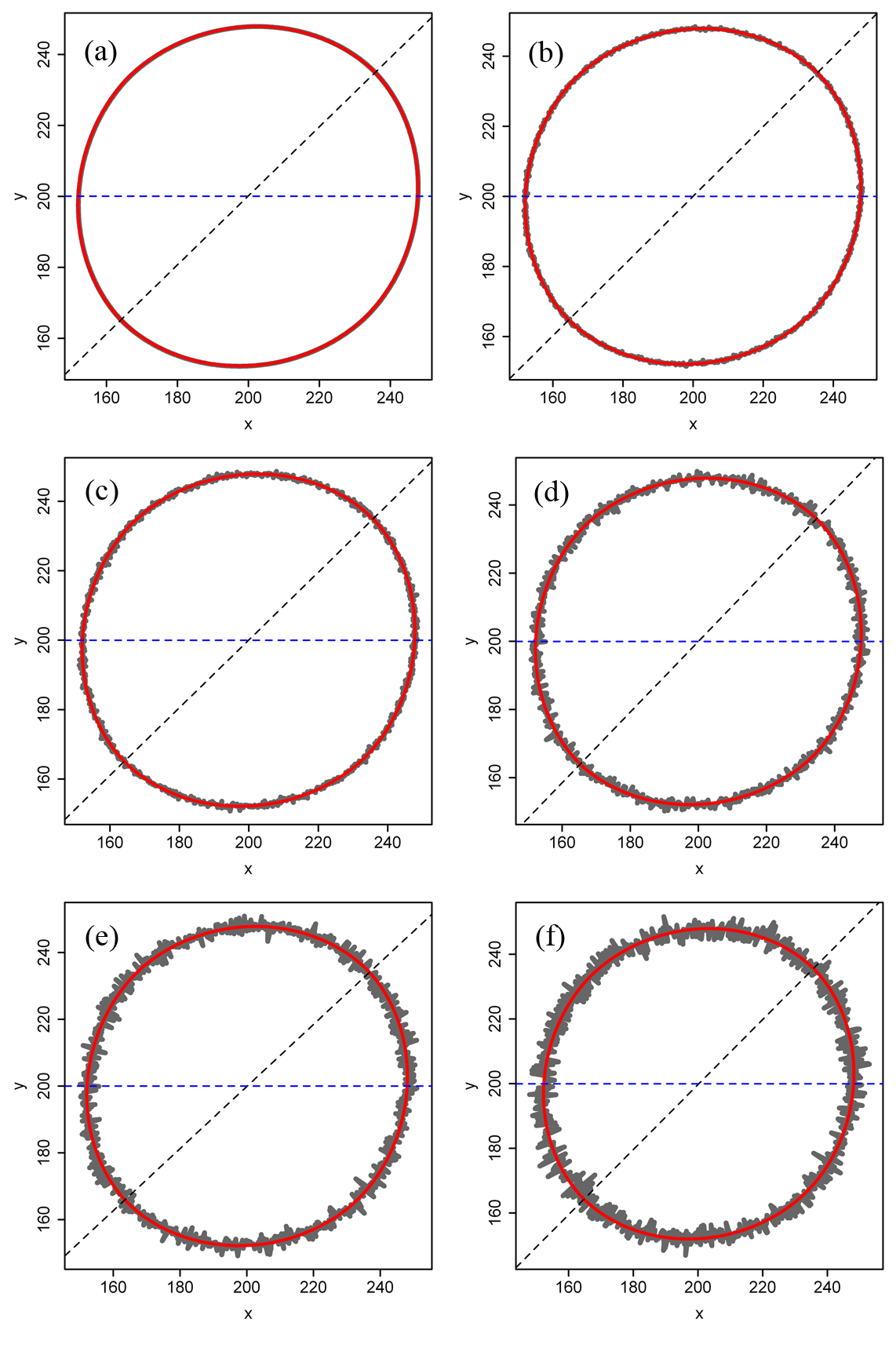

Supplement: Supplementary file 5 [file Image3.TIF]

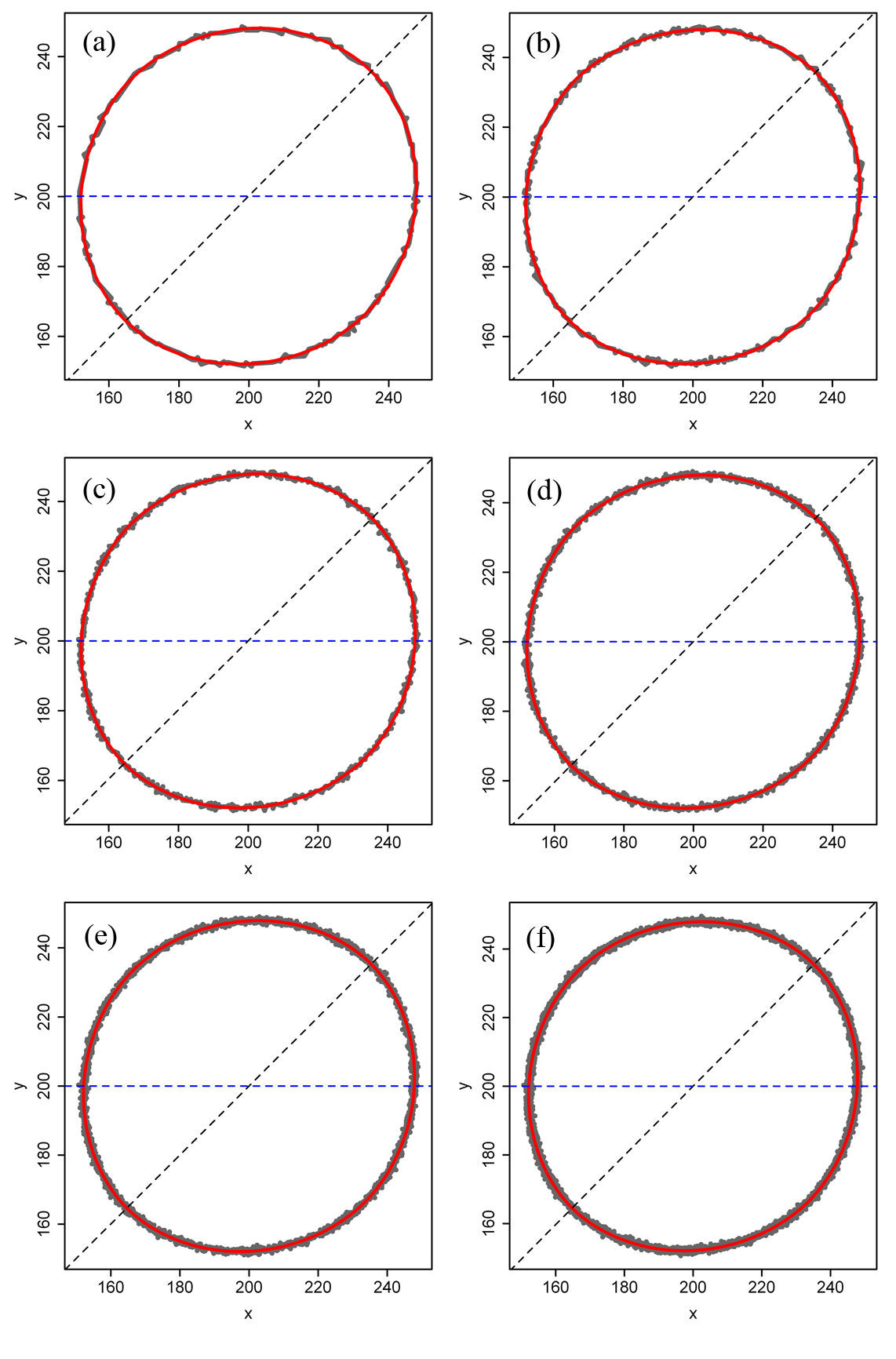

Supplement: Supplementary file 6 [file Image4.TIF]

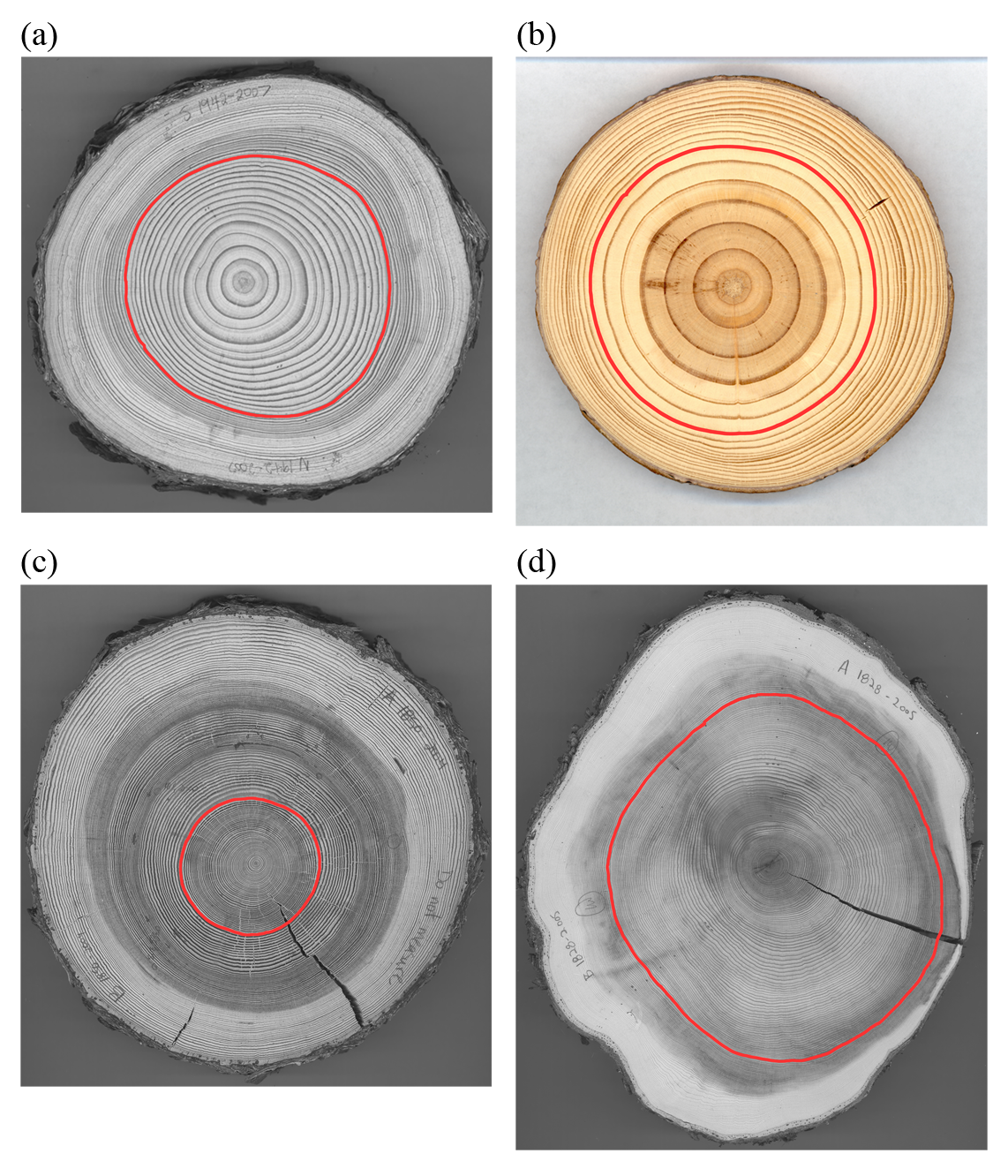

Supplement: Supplementary file 7 [file Image5.TIF]

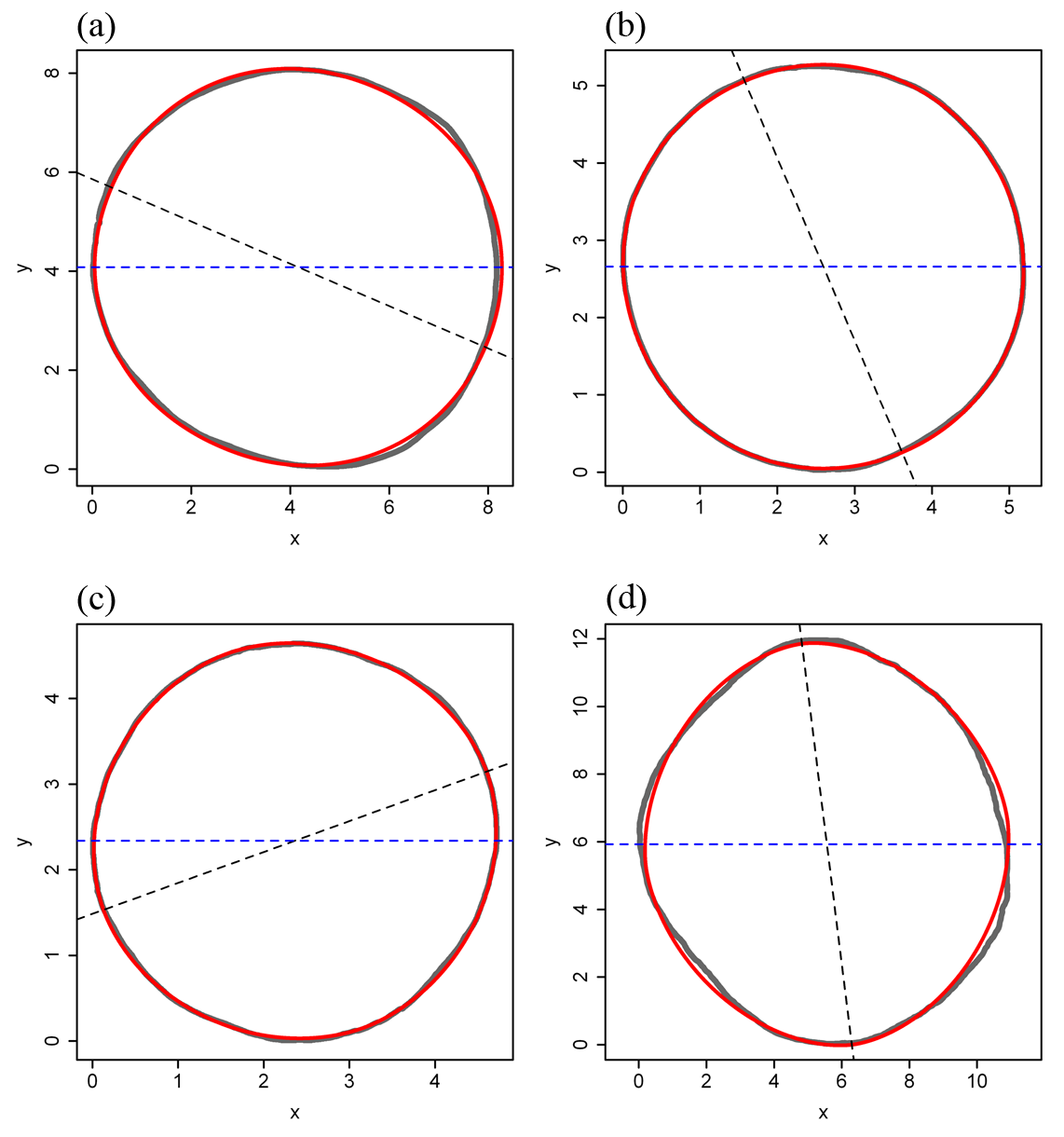

Supplement: Supplementary file 8 [file Image6.TIF]
