# Supplementary material for: Capturing spiral radial growth of conifers using the superellipse to model tree-ring geometric shape
Source: Front Plant Sci. 2015 Oct 15;6:856. doi: 10.3389/fpls.2015.00856 (PMC4606055; doi:10.3389/fpls.2015.00856)
Supplement: Supplementary file 2 [file DataSheet2.ZIP › Appendix S2. The usage of functions developed in the present study.doc]

**Appendix S2. The usage of functions developed in the present study**

**1. MATLAB Function Usage**

The user needs to scan the cross section with its tree rings. For any particular tree ring, the user can use the Magnetic Lasso Tool in **Adobe PhotoShop** (e.g., **PhotoShop CS2**) to create a new layer that only includes a particular tree ring. Then we extract the planar coordinates for the tree-ring boundary in the new image.

Xue (2009) proposed a **MATLAB** function (M-file) to extract the Cartesian coordinates of a scanned image in bmp (bitmap) format, but that function cannot automatically read the width and height of an image. To conveniently extract the Cartesian coordinates of a scanned tree-ring image, we substantially improved Xue’s original function and named it the “profile” function (also M-file) in **MATLAB** (version ≥ 6.5). This function can automatically read the planar coordinates of boundary, width, height, and resolution of a scanned image in bmp format. For other commonly used image formats, such as eps, jpg, gif, tif, etc., we can easily use **Adobe PhotoShop** to transform them to the bmp format. Fig. E1 shows a scanned tree ring randomly chosen from the first tree cross section of white spruce (*Picea glauca*, WS-1) in bmp format. The “profile” function requires that the outermost tree-ring boundary is included by the smallest rectangle in the image. In addition, to save running time, we recommend a resolution of 200 DPI for a bmp image for *a single tree ring* with 10 cm × 10 cm, 400 DPI for a bmp image with 5 cm × 5 cm, more than 800 DPI for a bmp image with 1 cm × 1 cm or less than this size.

Fig. E1. A scanned tree ring from the first tree cross section of white spruce sample WS-1 in bmp format. Note that the image boundary is exactly the smallest rectangle that includes the outermost boundary of tree ring. The image width is 8.97 cm, and the image height 8.71 cm. Image resolution is 400 DPI (dots per inch).

The “profile” function should be saved to the **MATLAB** working directory so it can be carried out. The user can also change the **MATLAB** working directory to use the “profile” function. Assume that the “profile” function has been stored into a folder named “tree.rings” on the C: drive. After opening MATLAB, we can type the code below on the command line to change the working directory to the “tree.rings” folder as follows:

cd 'c:\tree.rings\'

Assume that we have saved a bmp image named “SF” to the C: drive, and we wish to write the planar coordinates of the boundary extracted from the image to the C: drive as a table. Because MATLAB permits writing a csv (comma separated value) file, we will write a csv file named “edge_data” to the C: drive. Then we can carry out the “profile” on the command line as follows:

profile(1, 1, 'c:\SF.bmp', 'c:\edge_data.csv')

The first input item is used to control the ratio of the image width to the actual tree-ring width, the second input item is used to control the ratio of the image height to the actual tree-ring height, the third input item is used to read a bmp image from a particular directory, and the fourth input item is used to write a csv file to a particular directory. In the present case, we assume that the width ratio and the height ratio are both equal to 1 for convenience. After performing the function, the user can access the csv file on the C: drive that has stored the planar coordinates of the tree-ring boundary extracted from the image. The user can also obtain the figure based on the extracted boundary as shown in Fig. E2.

Fig. E2. The planar coordinates extracted from the tree-ring image

**2. R Function Usage**

For the present study, we developed ten **R** functions based on **R** software (version ≥ 3.0.0) (R Core Team, 2013) for the Microsoft Windows operating system: “simu.sf,” “optim.sf,” “fit.sf,” “area.sf,” “perc.sf,” “cv.sf,” “fit.logistic,” “angle.corr”, “perimeter.sf” and “optim.circle.” The software **R** can be freely downloaded from its website:

http://www.r-project.org/

Among these nine **R** functions, the “area.sf” and “perc.sf” functions depend on an **R** package of “splancs” (Rowlingson and Diggle, 1993), which can be installed online by typing the following code in the **R** Console:

install.packages("splancs")

The user can then discretionarily select a CRAN mirror to install it. After installing the package, we can save the nine functions to the working directory of **R**. The working directory of **R** can be changed by selecting the “Change dir…” option in the “File” menu. Assume that these nine functions are saved into a folder named “tree.rings” on the C: drive. To call these nine functions, we can use the following codes in the **R** Console:

wd <- c("c:/tree.rings/")

source(paste(wd, "simu.sf.R", sep=""))

source(paste(wd, "optim.sf.R", sep=""))

source(paste(wd, "fit.sf.R", sep=""))

source(paste(wd, "cv.sf.R", sep=""))

source(paste(wd, "area.sf.R", sep=""))

source(paste(wd, "perc.sf.R", sep=""))

source(paste(wd, "fit.logistic.R", sep=""))

source(paste(wd, "angle.corr.R", sep=""))

source(paste(wd, "perimeter.sf.R", sep=""))

Before using these **R** functions, we need to read the planar coordinates of tree-ring boundary extracted from the bmp image by the above **MATLAB** function (i.e., the “profile” function). Assume that these coordinates have been stored in a csv file named “edge_data” on the C: drive. We can read these data in the **R** Console by the following codes:

data	<- read.csv("c:/edge_data.csv", header=F)

x.provi <- data[,1]

y.provi <- data[,2]

x.provi and y.provi represent the x- and y-coordinates of tree-ring boundary, respectively.

2.1. *The “simu.sf” function*

The "simu.sf" function is used to produce the [Cartesian](http://en.wikipedia.org/wiki/Cartesian_coordinate_system) coordinates of a simulated tree ring that can be perfectly described by the superellipse equation, and the superellipse is actually a special case of the superformula equation proposed by Gielis (2003b). Here, “sf” is an abbreviation of *superformula*. The given parameters should include the abscissa and ordinate of the pole (*x*_0_, *y*_0_) in the Cartesian system, the angle θ between the major axis and the x-axis, the major semi-axis *a*, the ratio *k* of the minor semi-axis (*b*) to the major semi-axis (*a*), and the power *n*. Note that the parameters must be provided by an array (i.e., par). In addition, we also need to provide the coefficient of variation (i.e., CV) in the radial coordinate (*r*) in any a direction. Here, we assume that the CVs in all directions are identical for simplicity. And we also need to provide an array of the angular coordinates (i.e., phi). We show the usage of this function below:

x0.val	<- 200

y0.val	<- 200

theta.val <- pi/4

a.val	<- 50

k.val	<- 0.95

n.val	<- 1.90

Phi	<- seq(0, 2*pi, len=1000)

Para	<- c(x0.val, y0.val, theta.val, a.val, k.val, n.val)

CV.val	<- 0.01

res1	<- simu.sf(phi=Phi, par=Para, CV=CV.val, fig.opt = T)

x.simu	<- res1$x

y.simu	<- res1$y

After performing this function, it will produce x- (i.e., x.simu) and y-coordinates (i.e., y.simu) of a simulated tree ring. There is an option of “fig.opt.” If we choose “fig.opt = TRUE” or “fig.opt = T,” the simulated tree ring will be shown; otherwise, it will be not shown. Fig. E3 shows the simulated tree ring. In addition, this function can be also used to describe the tree ring when the abscissa and ordinate of the pole, the transferred angle, and the superellipse model parameters are known.

Fig. E3. A simulated tree ring by using the “simu.sf” function. The dark small open circles represent the data points from a simulated tree ring with CV of *r* = 1%. The dark dashed line represents the direction of major axis; and the two blue dashed lines represent the directions of x- and y-axis, respectively.

2.2. *The “optim.sf” function*

This function is used to fit the superellipse model parameters when the [Cartesian](http://en.wikipedia.org/wiki/Cartesian_coordinate_system) coordinates of a ring are know by using the “optim” function in **R**. We need provide the initial values of the abscissa and ordinate of the pole (i.e., x0.initial and y0.initial), the angle between the major axis and the x-axis (i.e., theta.initial), the major semi-axis (i.e., a.initial), the ratio of the minor semi-axis to the major semi-axis (i.e., k.initial), and the power (i.e., n.initial). The option "fig.opt" is used to determine whether to draw a figure. We show the usage of this function below:

x.width	<- range(x.provi)[2] - range(x.provi)[1]

y.width	<- range(y.provi)[2] - range(y.provi)[1]

a.ini	<- max(y.width, x.width)/2

x0.ini	<- (min(x.provi) + max(x.provi))/2

y0.ini	<- (min(y.provi) + max(y.provi))/2

theta.ini <- pi/4

k.ini	<- 0.95

n.ini	<- 1.90

res2	<- optim.sf(x.provi, y.provi, x0.ini, y0.ini, theta.ini, a.ini, k.ini,

n.ini, fig.opt="T", para.list="F", convergence="Chi.square")

Here, "res2" is only a name to save the running results, so the user can rename it. Note that the figure will exhibit a standardized data fitting. That means the abscissa and ordinate of the pole is (0, 0), and the angle between the major axis and the x-axis equals 0. The option of "para.list" is used to determine whether to display the fitted parameters on the screen in the **R** Console. After performing the function, it will produce the fitted parameters (par), the coefficient of determination (R.square), the χ^2^ (Chi.square) or the residual sum of squares (RSS) that depends on the user’s choice, the observed values of radial coordinates (r.obs), the predicted values of radial coordinates (r.pred), the observed x coordinates of the tree-ring boundary (x.obs), the predicted x coordinates of the tree-ring boundary (x.pred), the observed y coordinates of the tree-ring boundary (y.obs), and the predicted y coordinates of the tree-ring boundary (y.pred). The output items of r.obs, r.pred, x.obs, x.pred, y.obs and y.pred are also standardized. That is, the abscissa and ordinate of the pole is (0, 0) and the angle between the major axis and the x-axis equals 0. The user can check the output items by using names(res2) and call any an output item by res2$ + its corresponding name, e.g., res2$par. The option of “convergence” is used to choose the convergence condition for the “optim” function in **R**. We propose two methods: the lowest χ^2^ and the lowest RSS. In general, using the lowest χ^2^ can obtain a lower CV in the radial coordinate. Fig. E4 shows the data fitting by using the “optim.sf” function to the dataset of (x.provi, y.provi) obtained from using the “profile” function as mentioned above.

Fig. E4. Standardized data fitting by using the “optim.sf” function. The dark small open circles represent the data points in the tree ring with CV of *r* = 1%. Because the observed data points are so many, these circles appear to be a dark solid line. The red solid line represents the predicted ring.

2.3. *The “fit.sf” function*

Sometimes we hope to obtain the best fitted results from some combinations for different parameter initial values. We developed the “fit.sf” function to achieve this goal. We show the usage of this function below:

x0.range	<- x0.ini

y0.range	<- y0.ini

theta.range <- seq(0, pi/2, by=pi/8)

a.range	<- a.ini

k.range	<- seq(0.8, 1, by=0.1)

n.range	<- seq(1.8, 2.2, by=0.1)

res3	<- fit.sf(x.provi, y.provi, x0=x0.range, y0=y0.range,

theta=theta.range, a=a.range, k=k.range, n=n.range,

para.list=T, fig.opt=T, convergence="Chi.square")

The usage of this function is similar to that of the “optim.sf” function. However, the initial value(s) of any parameter can be an array or a single value. There is also an option of “fig.opt.” When we choose “fig.opt=T,” it will display the fitted results based on the transferred data points in a ring. That is, the abscissa and ordinate of the pole can be different from (0, 0), and the angle is not always 0 again. Fig. E5 shows the fitted results. Note the similarity and difference among Figs. E2, E4, and E5. In addition, the output items of this function are also the same as those of the “optim.sf” function. Here, we do not display them in detail.

In order to further improve the accuracy of parameter estimates, we can re-fit the data based on the forward step’s parameter estimates as the initial values of the new fitting:

res4	<- fit.sf(x.provi, y.provi, res3$par[1], res3$par[2],

res3$par[3], res3$par[4], res3$par[5], res3$par[6],

para.list=T, fig.opt=T, convergence="Chi.square")

Fig. E5. Data fitting to the scanned ring by using the superellipse equation. The gray solid line represents the observed tree ring; the red solid line represents the predicted ring. The dark dashed line represents the direction of major axis; the blue dashed line represents the direction of x-axis.

2.4. *The “cv.sf” function*

If the superellipse model can perfectly describe the shape of any tree ring, we expect that a very small difference between the observed and predicted radial coordinates in any direction. We assume that the coefficients of variation in all directions are identical. The “cv.sf” function is used to measure such a CV.

res5 <- cv.sf(res4$r.obs, res4$r.pred, fig.opt=T)

print(paste("CV = ", res5$r.cv*100, "%", sep=""))

We need to provide the observed and predicted radial coordinates, which can be obtained from using the “fit.sf” function. The option of “fig.opt” is used to determine whether to produce two relevant panels. The first panel is to show the probability density of the *r* residuals and the second panel is to show the comparison between the observed and predicted radial coordinates. Fig. E6 displays these two panels.

Fig. E6. Probability density of *r* residuals (a), and the comparison between the observed and predicted radial coordinates (b). In the left panel, the red solid line represents the theoretical normal probability density. In the right panel, the red solid line represents the straight line equation of *y* = *x*.

2.5. *The “area.sf” function*

This function is used to calculate the area encircled by the ring. Its usage is listed as follows:

\# Example 1:

res6 <- area.sf(res4$par[4:6], point.num=3000)

print(paste("Predicted area is", res6$pred.area))

Before carrying out this function, an array (or matrix) including the superellipse model parameters *a*, *k,* and *n* need be provided. “point.num” is used to define the number of points in a ring. A larger number of points in a ring can provide a more accurate estimate of the area. The function depends on the **R** package of “splancs” as mentioned above. In fact, this function was derived from the “areapl” function in the “splancs” package. Instead, we can directly use the “areapl” function to calculate the area encircled by the ring:

library(sp)

library(splancs)

shape1	<- cbind(x.provi, y.provi)

real.area <- areapl(shape1)

print(paste("Real area is", real.area))

shape2	<- cbind(res4$x.pred, res4$y.pred)

pred.area <- areapl(shape2)

print(paste("Predicted area is", pred.area))

Here, “real.area” denotes the area based on the scanned ring, and “pred.area” denotes the area based on the fitted parameters by using the “fit.sf” function. Although we can calculate the area by directly using the “areapl” function in the “splancs” package, it is in practice more convenient to use the “area.sf” function developed in the present study during calculating the confidence interval of area. The “area.sf” function supports a matrix input for parameters, which can be shown as follows:

\# Example 2:

par.matrix <- rbind(c(4.50, 0.95, 2.12), c(5.00, 1.00, 2.00))

area.sf(par.matrix)

2.6. *The “perc.sf” function*

This function is mainly used to calculate the confidence intervals (CIs) and standard errors of the fitted parameters, including the abscissa and ordinate of the pole (*x*_0_, *y*_0_), the angle between the major axis and the x-axis (θ), the superellipse model parameters (*a*, *k*, and *n*), the coefficient of determination (*R*^2^), the χ^2^ or the RSS, and the area encircled by the ring. The function was developed on the basis of the bootstrap percentile method (Efron and Tibshirani, 1994). The user can set more than 2000 bootstrap replications by changing the option of “boot.num” in the “perc.sf” function. We display the usage of this function below:

res7 <- perc.sf(x.provi, y.provi, boot.num=2000, point.num=3000,

ini.val=res3$par, alpha=c(0.50, 0.025, 0.975), fig.opt=T)

res7$bootmatrix

res7$ci.mat

Here, “x.provi” and “y.provi” are the planar coordinates of a given ring. “boot.num” is used to define the number of bootstrap replications. “point.num” is used to define the number of points in a ring for calculating the area encircled by that ring. “ini.val” as an array is used to provide the initial values of parameters. We propose using the fitted parameters by using the “fit.sf” function as the initial values of parameters, so we take “ini.val=res3$par” (**Note:** Here we did not choose “ini.val=res4$par”). “alpha” is used to set the percentiles. In the above example, we suggest three percentiles: 0.50, 0.025, and 0.975. The first can provide the medians of target parameters and the last two can provide 95% CIs of target parameters. The “fig.opt” option is used to determine whether to draw the histograms for four crucial parameters: the angle between the major axis and the x-axis (θ), the superellipse model parameters (*a*, *k*, and *n*). Fig. E7 displays the case of “fig.opt=T.” This function will produce two output items: bootmatrix and ci.mat. The user can call them by using res7$bootmatrix and res7$ci.mat. The former is a matrix that is used to store the fitted parameters corresponding to different bootstrap replications, and the latter is a matrix that is used to store the standard errors and the CIs of target parameters.

Fig. E7. Histograms of four parameters. The red solid lines represent the predicted probability densities.

2.7. *The “fit.logistic” function*

This function is used to fit the growth equation of the areas encircled by the tree rings with different ages. This function was derived from the early function developed in Shi et al. (2013, 2014). However, the convergence condition of the early function was only based on the lowest χ^2^. We provide two choices for the convergence condition: the lowest χ^2^ and the lowest RSS. The improved usage is listed as follows:

\# Building the logistic model

logistic <- function(P,x){

A.inf <- P[1]

alpha <- P[2]

beta <- P[3]

A.inf / (1+exp(alpha - beta*x))

}

\# Simulating an array of areas with different ages

age.val	<- seq(1, 100, by=1)

random.val <- c()

for (i in 1:length(age.val)){

random.val <- c(random.val, rnorm(1, 0, sd=1/20*age.val[i]))

}

area.val	<- logistic(c(250, 6, 0.15), age.val) + random.val

\# Using the "fit.logistic" function to fit the simulated data

res8	<- fit.logistic(age.val, area.val, A.inf.arr=seq(100, 500, len=10),

alpha.arr=1, beta.arr=0.1, fig.opt=TRUE, convergence="Chi.square")

res8

First, we built a logistic function. It requires inputting an array including three elements (i.e., the maximum asymptotic area *A*_∞_, the parameter α, and the growth rate β) and the predictors (i.e., ages). Second, we simulated an array of tree-ring areas aged 1 to 100. At every age, we assumed the CV in the observed area equals 5% of its real area. Third, we used the “fit.logistic” function to fit the simulated tree ring areas of different ages. “age.val” denotes the observed ages. “area.val” denotes the observed areas encircled by the tree rings of different ages. “A.inf.arr,” “alpha.arr,” and “beta.arr” are used to provide the initial values for carrying out a non-linear fitting. Each can be a single value or an array. “fig.opt” is used to determine whether to display the data fitting figure. The option of “convergence” is used to choose the convergence condition. It can be also “Chi.square” or “RSS.” After carrying out the “fit.logistic” function, the fitted model parameters in the logistic equation will be shown. The coefficient of determination (*R*^2^), the χ^2^, or the RSS will be also shown. Fig. E8 displays the fitted results.

Fig. E8. Fitted areas of tree rings of different ages. The small open circles represent the simulated areas encircled by the tree rings at different ages. The red solid line represents the predicted areas.

2.8. *The “angle.corr” function*

This function is used to correct the angle estimates for the tree rings at different ages from the same cross section. Because the superellipse is symmetrical around the major axis, an angle estimate might be its real angle θ or $\text{θ}\pm\text{π}$. Using these three angles in the superellipse equation can obtain the same fitted results. To explore whether there is a phenomenon of angle rotation of tree-ring major axis, it is necessary to transform $\text{θ}\pm\text{π}$ to θ. Also, there might be abnormal angle estimates because of the unclear tree-ring images. We hope to exclude these abnormal angle estimates during analyzing the angle rotation. This function was developed to achieve the aforementioned two goals. We show the usage of this function below:

\# To simulate a group of estimated angles y2

x1	<- seq(1, 315, by=1)

w	<- 1/50

y1	<- 2*pi*sin(w*x1)

y2	<- rep(NA, length(y1))

ind1 <- which( y1 >= 0 )

ind2 <- which( y1 < 0 )

y2.1 <- y1[ind1] - pi

y2.2 <- y1[ind1] + pi

y2.3 <- y1[ind1]

y2.4 <- y1[ind2] - pi

y2.5 <- y1[ind2] + pi

y2.6 <- y1[ind2]

y21 <- c()

y22 <- c()

for(i in 1:length(y2[ind1])){

ind3 <- sample(1:3, 1)

if(ind3==1) y21[i] <- y2.1[i]

if(ind3==2) y21[i] <- y2.2[i]

if(ind3==3) y21[i] <- y2.3[i]

}

for(i in 1:length(y2[ind2])){

ind3 <- sample(1:3, 1)

if(ind3==1) y22[i] <- y2.4[i]

if(ind3==2) y22[i] <- y2.5[i]

if(ind3==3) y22[i] <- y2.6[i]

}

y2[ind1] <- y21

y2[ind2] <- y22

\# To set eight abnormal values

y2[seq(6, 66, by=20)]	<- y2[seq(6, 66, by=20)] + pi/4

y2[seq(106, 166, by=20)] <- y2[seq(106, 166, by=20)] - pi/3

win.graph()

par(mar=c(5, 5, 1, 1))

plot(x1, y2, type="l", xlab="Year", ylab="Random angle")

\# To using the "angle.arr" function to correct the simulated angles

res9 <- angle.corr(x1, y2, auto.adj="TRUE", span=1/3, fold=5, fig.opt=TRUE)

Here, we simulated a group of angle estimates (y2), which came from the angle real values (y1) at different ages (x1). In this case, y2 might be y1 or y1 ± π that is actually randomly chosen from these three candidates. The crucial input items are age and angle in the function. The input option of “fig.opt” is used to determine whether to produce a figure that shows the corrected angles versus different ages. The input option of “auto.adj” is used to determine whether to automatically exclude the abnormal values of angle. These abnormal values are caused by fitting errors, especially by the unclear boundaries of the tree rings. In the function, we used the local regression method (i.e., the “loess” function in **R**; Cleveland and Grosse 1991) to find the abnormal values. The basic steps are: (i) use the “loess” function to provide the predicted values of angles, where the “span” option is used to control the degree of smoothing; (2) calculate the 25% and 75% quantiles for the absolute values of angle residuals; (3) determine whether an angle is abnormal based on whether the absolute value of its residual is outside of a given times the inter-quantile range (i.e., *the times (75% quantile – 25% quantile)*, where the times is denoted by “fold” in the function) apart from the lower or upper quantile. It is obvious that the defined “fold” should be related to the defined “span.” If a “span” is large, the “fold” should be small; otherwise, “fold” should be large. How to define a suitable combination of “span” and “fold” depends on the experience of users. After performing the function, it will produce the corrected angles (i.e., res9$y) and corresponding ages (i.e., res9$x). If we choose “auto.adj=T,” it will also produce the excluded angles (i.e., res9$y.exc) and ages (i.e., res9$x.exc), where these excluded data will be shown in a plot by small open circles with “X” symbols. Fig. E9 displays the corrected and excluded angles.

Fig. E9. Corrected angles between the major axis and the x-axis. Here, the small open circles represent the corrected angles. The red solid line represents the fitted values by using the local regression method. The small open circles with “X” symbol represent the excluded data by choosing “auto.adj=TRUE” with “span=1/3” and “fold=5.”

2.9. *The “perimeter.sf” function*

Assume a standard superellipse with the center in the origin (0, 0) and without an angle rotation (i.e., θ = 0). We can find that it is actually symmetrical around the origin. Thus, the whole perimeter is four times longer than the arc length of this superellipse in the first quadrant. We can easily calculate the arc length in the first quadrant by the arc length formula:

$$\int_{0}^{a} \sqrt{1+\left(y'\left(x\right)\right)^{2}}dx$$

where $y'\left(x\right)=-\frac{b}{a}\left(\frac{x}{a}\right)^{-1+n}\left(1-\left(\frac{x}{a}\right)^{n}\right)^{-1+\frac{1}{n}}$ and $b=ak$.

This function is used to calculate the perimeter(s) of tree ring(s). Its usage is listed as follows:

\# Example 1

res10	<- perimeter.sf(res4$par[4:6])

print(paste("Predicted perimeter is", res10$pred.perimeter))

\# Example 2

par.matrix <- rbind(c(4.50, 0.95, 2.12), c(5.00, 1.00, 2.00))

perimeter.sf(par.matrix)

Before carrying out this function, an array (or a matrix) including the superellipse model parameters *a*, *k*, and *n* need be provided. The predicted result(s) can be found by res10$pred.perimeter.

*2.10. The “optim.circle” function*

This function’s usage is similar to the “optim.sf” function. This function is mainly used to fit a circle equation’s parameter (i.e., the radius) when the [Cartesian](http://en.wikipedia.org/wiki/Cartesian_coordinate_system) coordinates of a ring (i.e., x and y) are know by using the “optim” function in **R**. We need provide the initial values of the abscissa and ordinate of the pole (i.e., x0.initial and y0.initial), and the radius (i.e., radius.initial). Here, these three parameters’ initial values can be omitted. The option "fig.opt" is used to determine whether to draw a figure. We show the usage of this function below:

res11 <- optim.circle(x.provi, y.provi, fig.opt="T", para.list="T", convergence="Chi.square")

res11

After carrying out this function, we can obtain the estimates of the coordinates of centre (i.e., res11$x0 and res11$y0) and the length of radius (i.e., res11$radius). Fig. E10 shows the data fitting by using the “optim.sf” function to the dataset of (x.provi, y.provi) obtained from using the “profile” function as mentioned above.

Fig. E10. Data fitting to the scanned ring by using the circle equation. The dark solid line represents the observed tree ring; the red solid line represents the predicted ring. The blue dashed line represents the direction of x-axis.

LITERATURE CITED IN THE APPENDIX S2

Cleveland, W. S. and Grosse, E. (1991). Computational methods for local regression. *Stat. Comput*. 1, 47–62. doi: 10.1007/BF01890836

Efron, B. and Tibshirani, R. J. (1994). *An introduction to the bootstrap*. New York: Chapman & Hall/CRC.

Gielis, J. (2003b). *Inventing the circle: the geometry of nature*. Antwerpen: Geniaal Press.

R Development Core Team. (2013). *R: a language and environment for statistical computing*. Vienna: R Foundation for Statistical Computing. Available at: http://www.R-project.org/

Rowlingson, B. S., Diggle P. J. (1993). Splancs: spatial point pattern analysis code in S-Plus. *Comput. Geosci.-UK* 19, 627–655. doi: 10.1016/0098-3004(93)90099-Q

Shi, P. J., Ishikawa, T., Sandhu, H. S., Hui, C., Chakraborty, A., Jin, X. S., et al. (2014). On the 3/4-exponent von Bertalanffy equation for ontogenetic growth. *Ecol. Model.* 276, 23-28. doi: 10.1016/j.ecolmodel.2013.12.020

Shi, P. J., Men, X. Y., Sandhu, H. S., Chakraborty, A., Li, B. L., Ou-Yang, F., et al. (2013). The “general” ontogenetic growth model is inapplicable to crop growth. *Ecol. Model.* 266, 1-9. doi: 10.1016/j.ecolmodel.2013.06.025

Xue, S.F. (2009). MATLAB application in planar graph boundary information extraction. *Journal of Hengshui University* 11(4), 25–27. (In Chinese with English Abstract)
